# iPSC-derived myelinoids to study myelin biology of humans

**DOI:** 10.1016/j.devcel.2021.12.009

**Published:** 2022-01-10

**Authors:** Owen G. James, Bhuvaneish T. Selvaraj, Dario Magnani, Karen Burr, Peter Connick, Samantha K. Barton, Navneet A. Vasistha, David W. Hampton, David Story, Robert Smigiel, Rafal Ploski, Peter J. Brophy, Charles ffrench-Constant, David A. Lyons, Siddharthan Chandran

(Developmental Cell *56*, 1346–1358.e1–e6; May 3, 2021)

In the originally published version of this article, in the “Generation of iPSC myelinoids” paragraph of the STAR Methods section, the authors inadvertently included typographical errors that reflected an internal error in transcription of methods. This has now been corrected in the article online, and the authors regret the error.

