# Supplementary material for: iPSC-derived myelinoids to study myelin biology of humans
Source: Dev Cell. 2021 May 3;56(9):1346–1358.e6. doi: 10.1016/j.devcel.2021.04.006 (PMC8098746; doi:10.1016/j.devcel.2021.04.006)
Supplement: Document S1. Figures S1–S5 [file mmc1.pdf]

Developmental Cell, Volume *56*

Supplemental information

iPSC-derived myelinoids to study myelin biology of humans

Owen G. James, Bhuvaneish T. Selvaraj, Dario Magnani, Karen Burr, Peter Connick, Samantha K. Barton, Navneet A. Vasistha, David W. Hampton, David Story, Robert Smigiel, Rafal Ploski, Peter J. Brophy, Charles ffrench-Constant, David A. Lyons, and Siddharthan Chandran

Figure S1: Characterisation and reproducibility of myelinoids, related to Figure 1

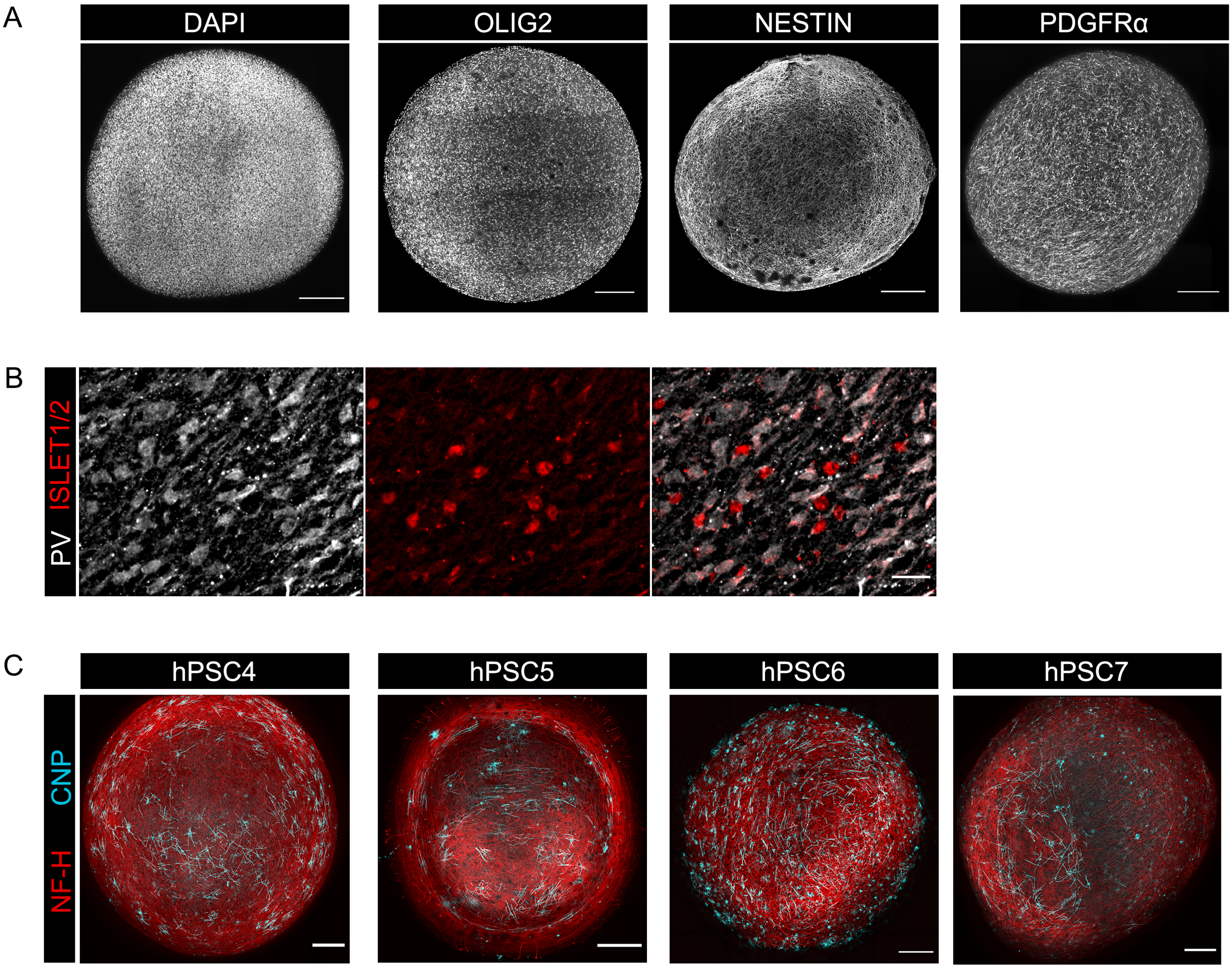

Figure S1: Characterisation and reproducibility of myelinoids, related to Figure 1.

A) Representative images of DAPI, OLIG2, NESTIN and PDGFRα at MI-0 in whole-mounted myelinoids (scale bar: 250μm).
B) Immuno-staining of PV and ISLET1/2 at MI-12 shows no colocalisation between these markers (scale: 25μm).
C) Representative images of CNP and NF-H-stained myelinoids at MI-12 from four additional cell-lines (scale bar: 250μm).

Figure S2: Distribution of myelin in iPSC myelinoids, related to Figure 2

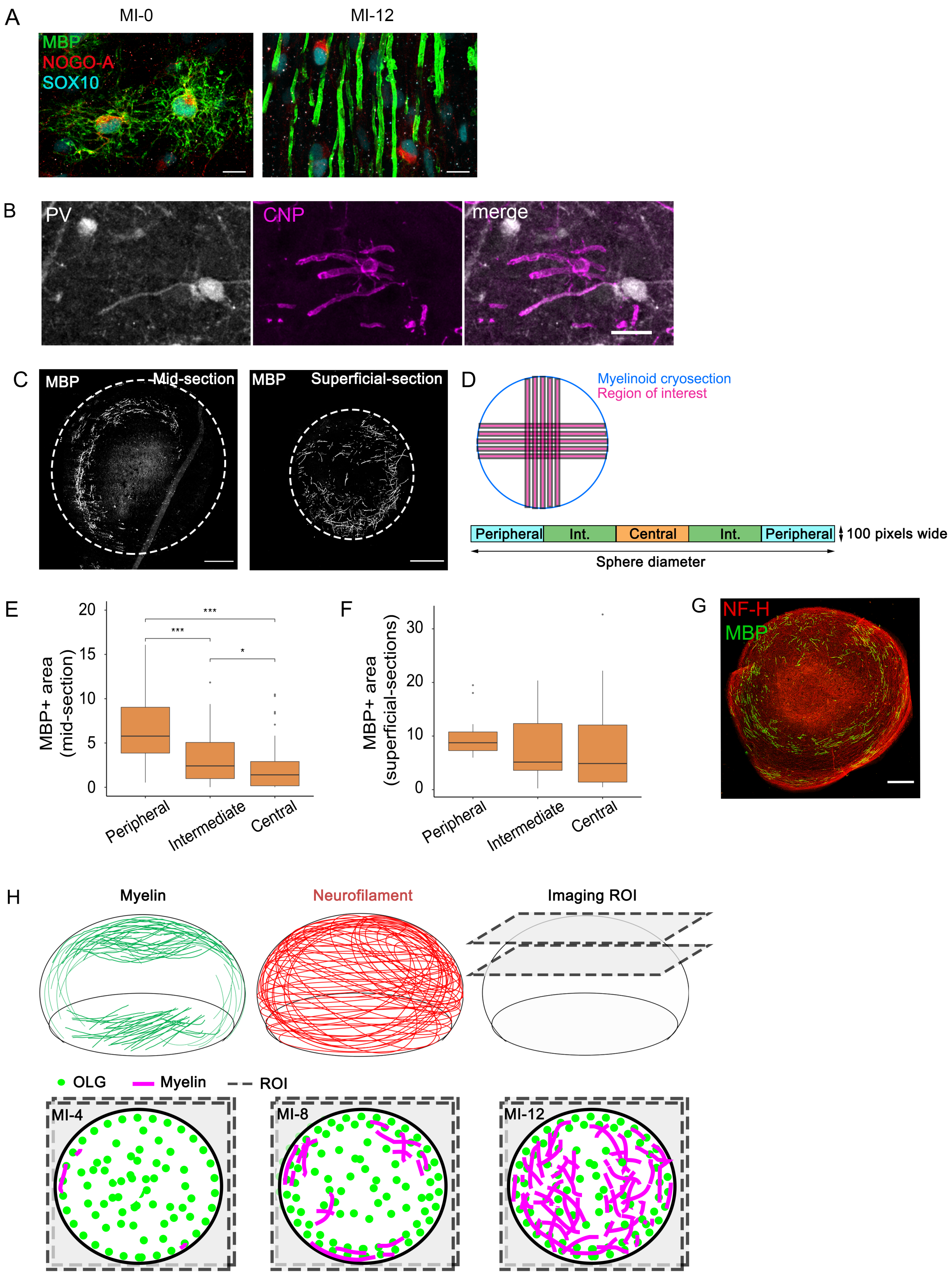

Figure S2: Distribution of myelin in iPSC myelinoids, related to Figure 2.

A) Mature oligodendrocytes stained for MBP, SOX10 and NOGO-A at MI-0 and MI-12 (scale bar: 10μm).
B) Immuno-staining of PV and CNP at MI-12 shows that individual oligodendrocytes can myelinate both PV+ and PV- axons simultaneously (scale bar: 25 μm).
C) Representative images of MBP+ myelin from a middle and superficial section of MI-12 myelinoids shows that myelin is predominantly found in the peripheral regions of mid-sectioned myelinoids and distributed across the entire area of the superficially-sectioned myelinoids (dashed line indicates section perimeter), scale bar: 250 μm.
D) Schematic overview for quantifying the distribution of MBP+ pixels across myelinoid cryosections. Multiple vertical and horizontal regions of interest were acquired across each section before images were thresholded and MBP+ pixels were measured as a function of the length of the ROI. Each ROI was then binned into peripheral, intermediate (Int.) and central regions for analysis (Figure S2E-F).
E-F) Quantification of MBP pixel area across middle and superficial myelinoid sections revealed 3.75-fold higher MBP+ pixel area in the periphery of sections compared to the central area (95% CI: 2.27- to 6.19-fold; $p < 0.001$); glmm with dummy variables for each region. $n = 111$ ROIs across 37 sections from 5 myelinoids from different batch-conversions.
G) Representative image of a mid-section myelinoid at MI-12 stained with MBP and NF-H shows myelination of axon tracts running around the periphery (scale bar: 250 μm).
H) Schematic depiction of the distribution of myelin and axons across iPSC myelinoids. The region of interest captured during whole-mounted imaging of myelinoids and schematic representation of myelin visualised within this ROI at MI-4, MI-8, and MI-12.

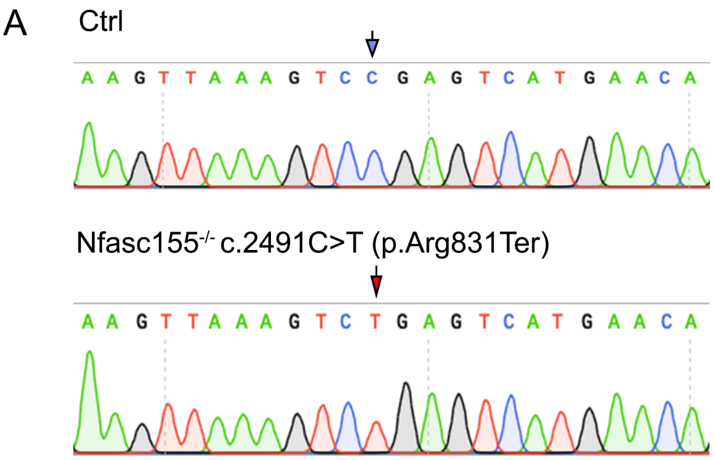

Figure S3: Sanger sequencing of NFASC gene, related to Figure 4.

A) Sanger sequencing of NFASC gene in control and Nfasc155-/- iPSCs, red arrow indicates nonsense mutation site.

Figure S4: Automated analysis of myelin distribution, related to Figure 5

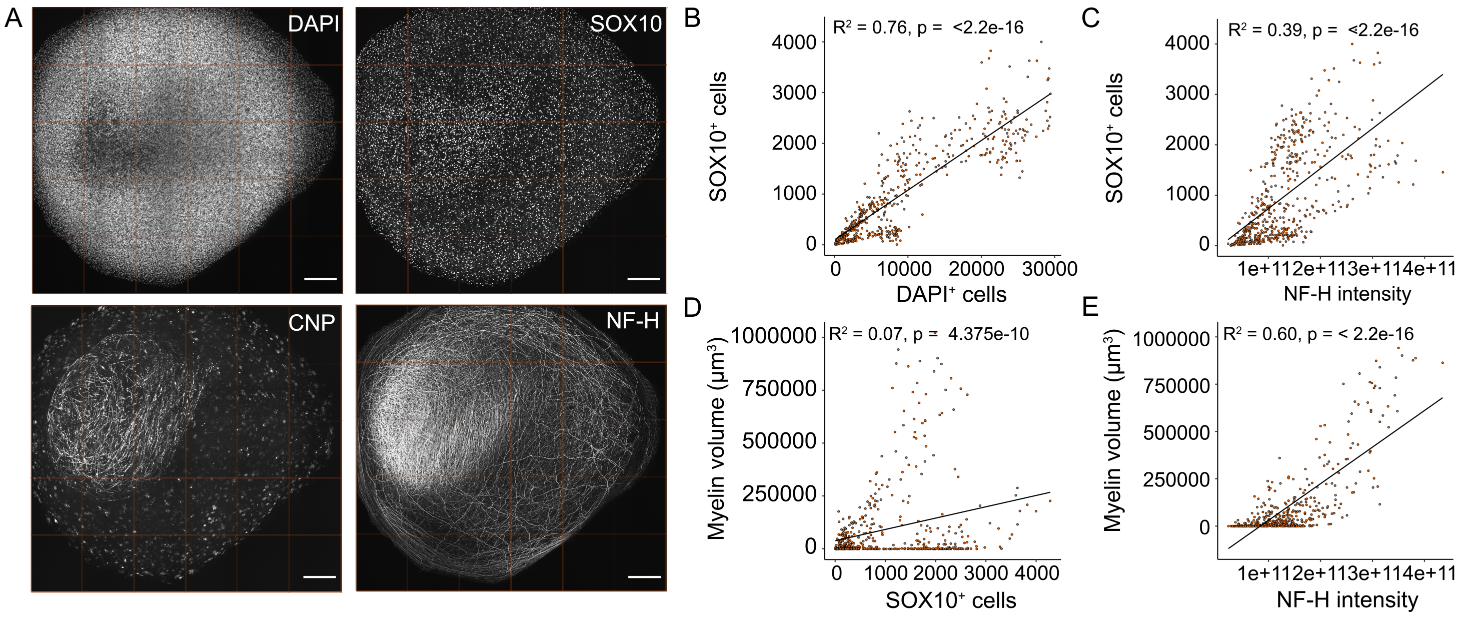

Figure S4: Automated analysis of myelin distribution, related to Figure 5.

A) Representative images of DAPI, SOX10, CNP and NFH staining from the same MI-12 myelinoid. Square grids indicate that each image is composed of 30 individual images tiled together. Correlation analysis performed on the small individual images was performed, scale bar: 250 µm.
B) Correlation of SOX10+ and DAPI+ cells, $R^2 = 0.76$, p = <2.2e-16.
C) Correlation of SOX10+ cells with NF-H intensity, $R^2 = 0.39$, p = <2.2e-16.
D) Correlation of myelin volume with SOX10+ cells, $R^2 = 0.07$, p = <4.38e-10.
E) Correlation of myelin volume with NF-H intensity, $R^2 = 0.6$, p = <2.2e-16.
Over 500 data points were analysed from 28 myelinoids at MI-12 across 6 conversions (Linear model with robust standard errors).

Figure S5: TeNT does not affect oligodendrocyte morphology cell-autonomously, related to Figure 6

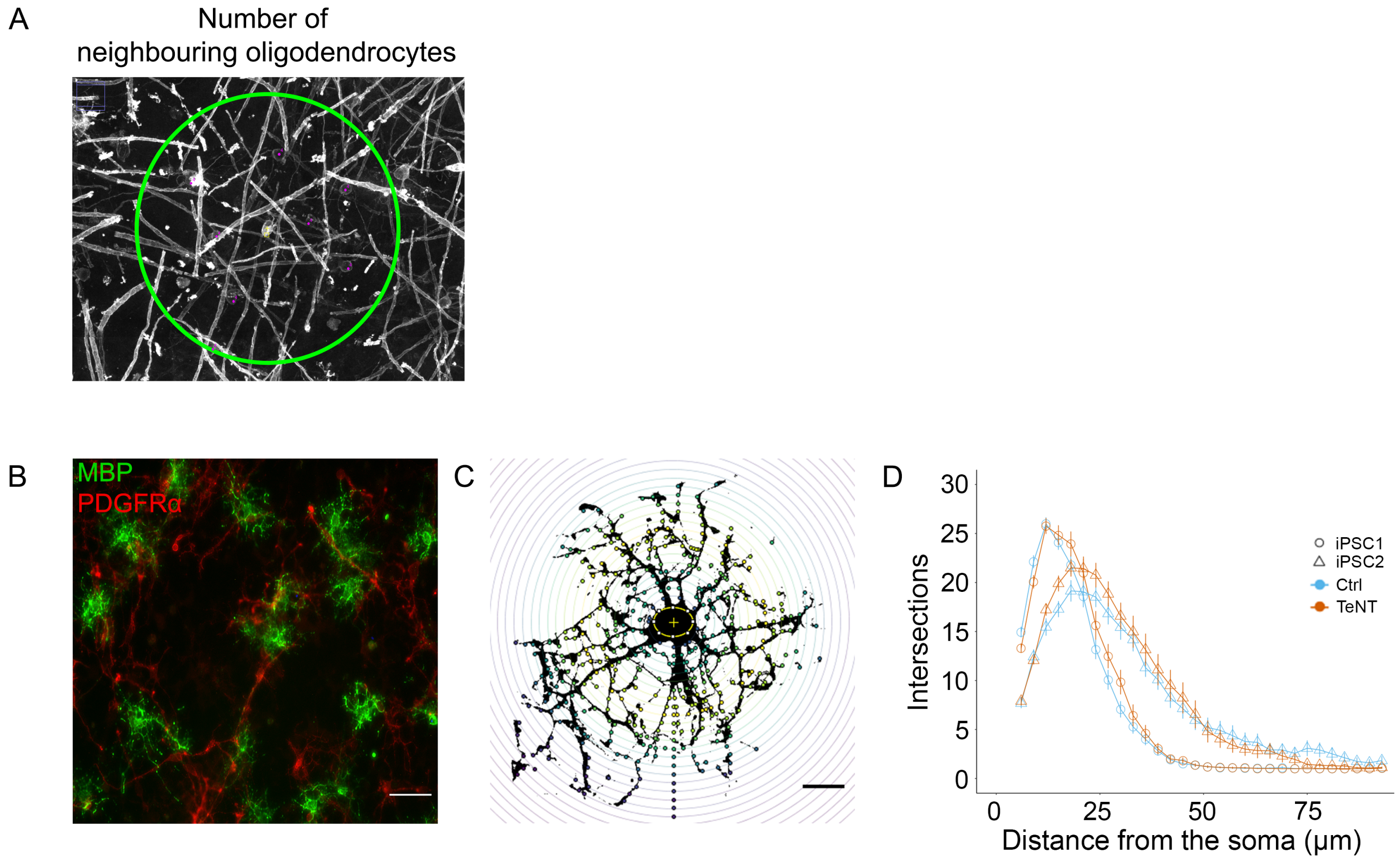

Figure S5: TeNT does not affect oligodendrocyte morphology cell-autonomously, related to Figure 6.

A) Nearest neighbour analysis was performed by drawing a 100 μm radius circle around individual cells and counting the number of myelinating oligodendrocytes within.
B) Representative images of MBP+ oligodendrocytes and PDGFRα+ OPCs from dissociated MI-0 myelinoids at day 14 (scale bar: 25μm).
C) Representative image of Sholl analysis on an MBP+ oligodendrocyte (scale bar: 10 μm; sholl radii emanating from the cell body (coloured) in 3 μm steps).
D) Sholl analysis of day 14 MBP+ oligodendrocytes from two different cell-lines treated with TeNT. Statistical modelling was performed as described by Wilson et al. (2018), J Neurosci Methods, glmm with radius and treatment as fixed effects and cell-line and batch-conversion as random effects. BH adjusted p values show no significant differences at any radii, n=84 cells (Ctrl) and 80 cells (TeNT) across two cell-lines and 3 batch-conversions each.
