# Supplementary material for: iPSC-derived myelinoids to study myelin biology of humans
Source: Dev Cell. 2021 May 3;56(9):1346–1358.e6. doi: 10.1016/j.devcel.2021.04.006 (PMC8098746; doi:10.1016/j.devcel.2021.04.006)
Supplement: Document S2. Article plus supplemental information [file mmc2.pdf]

Developmental Cell

Technology

iPSC-derived myelinoids to study myelin biology of humans

Graphical abstract

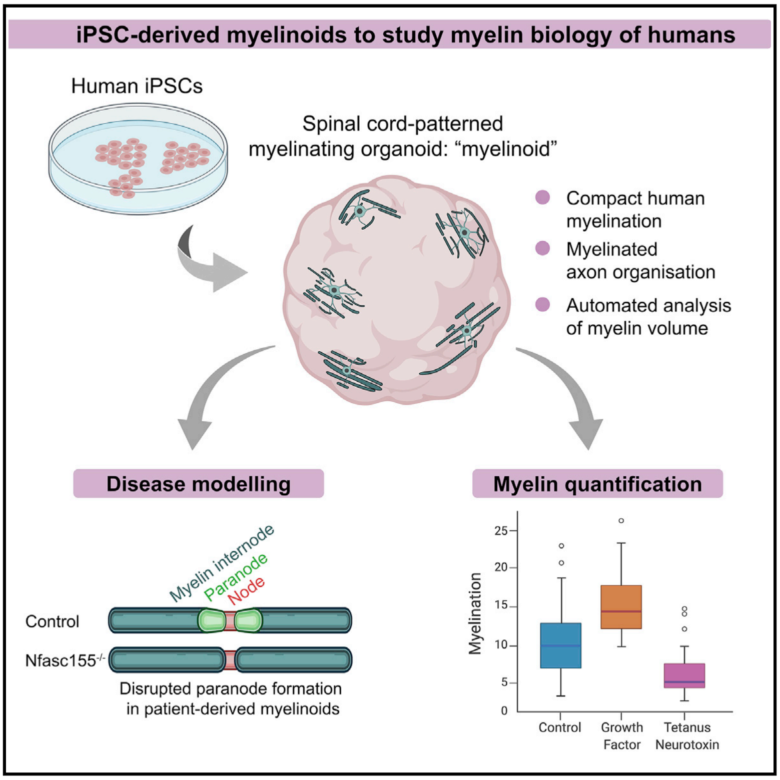

Authors

Owen G. James,
Bhuvaneish T. Selvaraj,
Dario Magnani, ...,
Charles ffrench-Constant,
David A. Lyons, Siddharthan Chandran

Correspondence

siddharthan.chandran@ed.ac.uk

In brief

James et al. describe a human stem cell-derived quantitative model of compact myelination that recapitulates structural organization of myelinated axons. They show impaired paranode formation using patient-derived cells, develop methods to analyze myelinating oligodendrocyte morphology and global myelination, and demonstrate hypomyelination in response to reduced synaptic vesicle release.

Highlights

- A human stem cell-derived organoid model of widespread and compact myelination
- Recapitulated impaired paranode formation in patient-derived cultures
- Pharmacological modulation of oligodendrocyte morphology and global myelin levels
- Blocking synaptic vesicle release leads to hypomyelination

James et al., 2021, Developmental Cell *56*, 1346–1358
May 3, 2021 © 2021 The Authors. Published by Elsevier Inc.
https://doi.org/10.1016/j.devcel.2021.04.006

CellPress

CellPress
OPEN ACCESS

Developmental Cell

Technology

iPSC-derived myelinoids to study myelin biology of humans

Owen G. James,[1,2,3,4] Bhuvaneish T. Selvaraj,[1,2,3] Dario Magnani,[1,2,3] Karen Burr,[1,2,3] Peter Connick,[1,2,4] Samantha K. Barton,[1,2,3,5] Navneet A. Vasistha,[2,3,6] David W. Hampton,[1,2,3] David Story,[1,2,3] Robert Smigiel,[7] Rafal Ploski,[8] Peter J. Brophy,[9] Charles ffrench-Constant,[10] David A. Lyons,[9] and Siddharthan Chandran[1,2,3,4,11,12,*]

[1]UK Dementia Research Institute at the University of Edinburgh, Edinburgh EH16 4SB, UK
[2]Centre for Clinical Brain Sciences, University of Edinburgh, Edinburgh EH16 4SB, UK
[3]Euan MacDonald Centre for Motor Neurone Disease Research University of Edinburgh, Edinburgh EH16 4SB, UK
[4]Anne Rowling Regenerative Neurology Clinic, University of Edinburgh, Edinburgh EH16 4SB, UK
[5]Florey Institute of Neuroscience and Mental Health, University of Melbourne, Melbourne, Australia
[6]Biotech Research and Innovation Centre, Copenhagen N 2200, Denmark
[7]Department of Pediatrics and Rare Disorders, Wroclaw Medical University, Wrocław 51-618, Poland
[8]Department of Medical Genetics, Medical University of Warsaw, Warsaw 02-106, Poland
[9]Centre for Discovery Brain Sciences, University of Edinburgh, Edinburgh EH16 4SB, UK
[10]MRC Centre for Regenerative Medicine, University of Edinburgh, Edinburgh EH16 4UU, UK
[11]Centre for Brain Development and Repair, inStem, Bangalore 560065, India
[12]Lead contact
*Correspondence: siddharthan.chandran@ed.ac.uk
https://doi.org/10.1016/j.devcel.2021.04.006

SUMMARY

Myelination is essential for central nervous system (CNS) formation, health, and function. Emerging evidence of oligodendrocyte heterogeneity in health and disease and divergent CNS gene expression profiles between mice and humans supports the development of experimentally tractable human myelination systems. Here, we developed human iPSC-derived myelinating organoids (“myelinoids”) and quantitative tools to study myelination from oligodendrogenesis through to compact myelin formation and myelinated axon organization. Using patient-derived cells, we modeled a monogenetic disease of myelinated axons (Nfasc155 deficiency), recapitulating impaired paranodal axo-glial junction formation. We also validated the use of myelinoids for pharmacological assessment of myelination—both at the level of individual oligodendrocytes and globally across whole myelinoids—and demonstrated reduced myelination in response to suppressed synaptic vesicle release. Our study provides a platform to investigate human myelin development, disease, and adaptive myelination.

INTRODUCTION

The formation and maintenance of myelin in the central nervous system (CNS) of humans is a dynamic life-long process (Westlye et al., 2010; Williamson and Lyons, 2018). Its disruption is associated with conditions across the life course including neurodevelopmental, psychiatric, and degenerative disorders (Ameis and Catani, 2015; Back, 2017; Huang et al., 2015; Kang et al., 2013; Kelly et al., 2018; Nasrabady et al., 2018; Noseworthy et al., 2000; Wolf et al., 2021). Critically, myelinated axon function requires both myelin wrapping and organization of distinct axonal subdomains, such as the paranodal axo-glial junction (PNJ) and the node of Ranvier. Disruption to either myelination or myelinated axon organization can lead to disease (Back, 2017; Lubetzki et al., 2020; Smigiel et al., 2018; Wolf et al., 2021). In addition to enabling efficient nerve conduction (Seidl, 2014) and providing metabolic support to axons (Simons and Nave, 2015), accumulating experimental evidence has led to the concept of adaptive myelination: changes in myelin structure in response to neuronal activity (Gibson et al., 2014; Mensch et al., 2015; Mitew et al., 2018). Although human imaging studies have revealed changes in white matter structure following visuomotor skill learning (Sampaio-Baptista and Johansen-Berg, 2017), direct cellular evidence of adaptive myelination by human oligodendrocytes has not yet been shown. Recent studies have also revealed previously unappreciated heterogeneity in human oligodendrocytes in health and disease as well as differences in gene expression profiles compared with mammalian model organisms, including mouse (Castelijns et al., 2020; Hodge et al., 2019; Jäkel et al., 2019; Xu et al., 2018). Together, this indicates that development of human models of myelination that could recapitulate structural and physiological features of myelin would serve as important experimental platforms to both better understand human myelin biology and complement existing models. Recently, human models of oligodendrogenesis have been reported that demonstrate oligodendrocyte differentiation in three-dimensional spheroids also containing neurons and astrocytes (Kim et al., 2019; Madhavan et al., 2018; Marton et al., 2019). These models have

 © 2021 The Authors. Published by Elsevier Inc.
This is an open access article under the CC BY license (http://creativecommons.org/licenses/by/4.0/).

been used to validate the effects of pro-differentiating compounds and for transcriptional comparison to primary tissue cells. However, while axonal ensheathment is evident in these models, structural organization of paranodal and nodal domains is not recapitulated (Madhavan et al., 2018). In the CNS, nodal assembly is dependent on PNJ formation, which occurs following ensheathment and coincides with myelin compaction (Rasband and Peles, 2015; Susuki et al., 2013; Wiggins et al., 1988). Nodal organization thus represents the formation of functionally mature myelinated axons. With respect to oligodendrocyte heterogeneity and the practicality of generating *in vitro* systems with mature myelinated axons, it is noteworthy that studies so far have focused on generating myelinating oligodendrocytes within forebrain-patterned cultures. There is increasing evidence that the regional identity of developing oligodendrocytes may influence their mature myelinating properties (Bechler et al., 2018) and myelination of the cortex, particularly of humans is a protracted process, potentially slowing down experimental interrogation (Westlye et al., 2010). For example, MBP$^+$ forebrain-derived oligodendrocytes are identified around 17–20 gestational weeks (gw) *in utero* but emerge several months before the onset of cortical myelination (Back et al., 2002; Jakovcevski et al., 2009). In contrast, the emergence of ventral spinal cord-derived oligodendrocytes occurs as early as 10 gw and is followed by the appearance of myelinated fibers up to 4 weeks later (Weidenheim et al., 1993). It would follow that spinal cord-patterned organoids might similarly develop mature myelinated axons at a faster rate than those patterned to the forebrain. Thus, we aimed to establish an induced pluripotent stem cell (iPSC)-derived, spinal cord-patterned model of myelin formation that enabled investigation of myelin development, disease, pharmacological interventions, and interrogation of activity-regulated myelination in a human context.

Design

To date, forebrain-patterned organoids demonstrate sparse myelination and lack appropriately organized myelinated axon architecture, limiting their use in the mechanistic evaluation of human myelin physiology in health and disease. Given the elongated gestation time of humans, modeling cortical myelin development *in vitro* has been particularly challenging. However, spinal cord myelination precedes cortical myelination in all vertebrates, and in humans by several months (Weidenheim et al., 1993). Therefore, to develop a robust iPSC-derived *in vitro* model of myelination, we sought to generate organoids with a ventral caudal cell fate as the pMN domain of the ventral spinal cord is the earliest site of oligodendrogenesis. Furthermore, we developed an automated analysis platform to quantify widespread compact myelin within myelinoids.

RESULTS

Generation and characterization of iPSC-derived myelinating organoids

In order to generate human myelinating cultures, we adapted our previously published protocol for generating iPSC-derived oligodendrocytes (Livesey et al., 2016; Magnani et al., 2019) to induce maturation of oligodendrocytes in spinal cord-patterned, three-dimensional organoids. Briefly, embryoid bodies generated from dual-SMAD inhibition were exposed to retinoic acid (RA) and smoothened agonist (SAG) to promote caudalization and ventralization, respectively, before being treated with PDGF-AA, IGF-1, and T3 to induce oligodendrogenesis (Livesey et al., 2016). Resulting spheroids contained an abundance of OLIG2$^+$ cells, demonstrating acquisition of pMN domain-derived cells as well as NESTIN$^+$ neural precursor cells, PDGFRα^+ oligodendrocyte progenitor cells (OPCs), CNP$^+$ oligodendrocytes, NF-H$^+$ neurons, and GFAP$^+$ astrocytes (Figures 1B and S1A). To promote myelin formation, 3 weeks following glial specification, individual spheroids were transferred onto hydrophilic PTFE-coated cell culture inserts (Figure 1A) to ensure efficient gas exchange at the air-liquid interface (Stoppini et al., 1991) and were maintained in myelination medium. We refer to this step as myelin induction (MI)-0. Organoids were further cultured and whole mounted or sectioned for immunocytochemical analysis at 4, 8, and 12 weeks post-MI-0 (MI-4, MI-8, and MI-12), which revealed widespread areas of axonal ensheathment by CNP$^+$ cells at MI-12 (Figure 1C). To characterize the regional identity and cellular composition of these cultures, qRT-PCR of rostrocaudal axis genes and immunostaining was next undertaken. High expression of HOXB5 and HOXB8 was demonstrated, consistent with a spinal cord identity (Dasen and Jessell, 2009; Nordström et al., 2006; Philippidou et al., 2012). Analysis of neuronal subtypes at MI-12 revealed the presence of ChAT$^+$ motor neurons and PV$^+$ ISLET1/2$^-$ interneurons, demonstrating differentiation of region-specific cell types (Figures 1D–1G and S1B) (Alvarez et al., 2005; Hossaini et al., 2011). Reproducibility of this protocol to generate myelinating organoids from multiple cell lines is demonstrated in Figure S1C.

Temporal development of myelin formation

To begin to analyze the temporal development of myelination, organoids were stained with CNP at MI-0, MI-4, MI-8, and MI-12. Whole-organoid imaging revealed an increase in the number of CNP$^+$ oligodendrocytes between MI-0 and MI-4, followed by increased CNP$^+$ sheath formation, suggestive of axonal ensheathment, from MI-8 onward (Figure 2A). Structural analysis of individual CNP$^+$ oligodendrocytes revealed maturation of cell morphology from having primarily thin, highly branched non-myelinating processes at MI-0 and MI-4 (Figures 2Bi and 2Bii), to the formation of distinct myelin sheaths from MI-8 onward (Figures 2Biii and 2Biv). Using SOX10, MBP, and CNP to respectively label oligodendroglial lineage, myelin sheaths and the cell body and processes of mature oligodendrocytes (Figure 2C), we found that the proportion of mature oligodendroglial cells (MBP$^+$CNP$^+$SOX10$^+$/SOX10$^+$), averaged across three cell lines, was stable between MI-4 and MI-12 (Figure 2D). However, the proportion of MBP$^+$ oligodendrocytes that engaged in myelin formation increased on average from 18% at MI-4 to 69% at MI-12 (Figure 2E). Mature oligodendrocytes were further identified by the co-expression of SOX10 and NOGO-A (Figure S2A). Immunostaining of MBP, NF-H, and MAP2 revealed that myelination was only observed on axons and not dendrites (Figure 2F), and temporal analysis of MBP$^+$ myelin sheaths showed increasing sheath lengths over time (Figure 2G). We found that myelin was targeted to both ChAT$^+$ and PV$^+$ axons and that individual oligodendrocytes could myelinate both PV$^-$ and PV$^+$ axons simultaneously, as was recently shown in the rodent neocortex

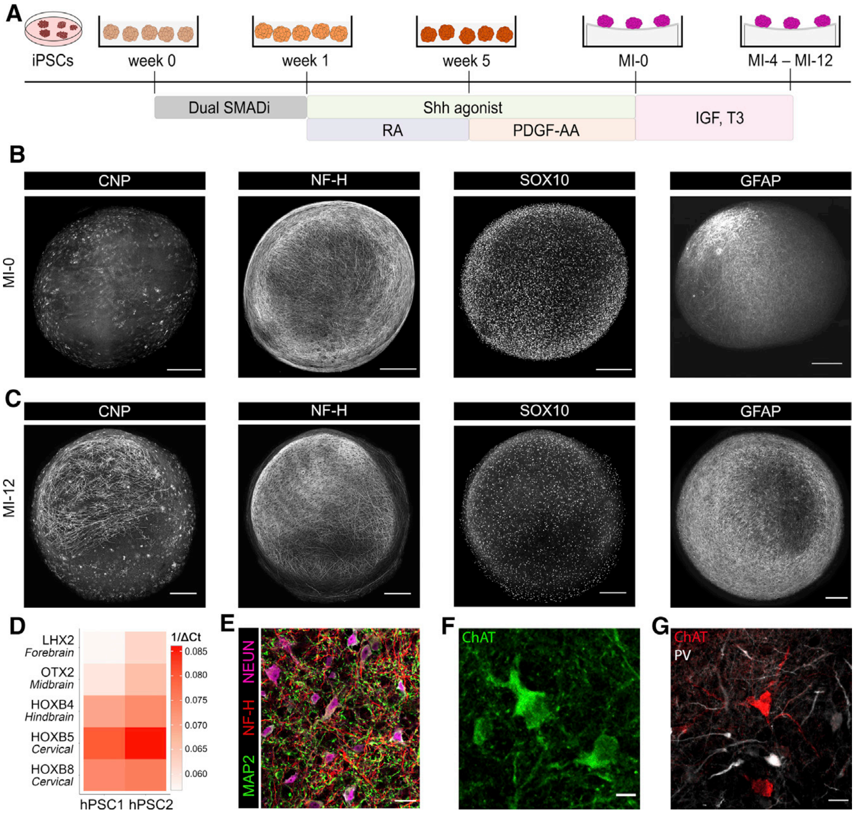

Figure 1. Generation and characterization of iPSC myelinoids

(A) Schematic of protocol for generating spinal cord-patterned organoids.

(B) Representative images of CNP^+ oligodendrocytes, NF-H^+ axons, $SOX10^+$ oligodendroglial cells and $GFAP^+$ astrocytes at MI-0 (scale bar, 250 μm).

(C) Representative images of CNP^+ oligodendrocytes, NF-H^+ axons, $SOX10^+$ oligodendroglial cells and $GFAP^+$ astrocytes at MI-12 (scale bar, 250 μm).

(D) Heatmap of qRT-PCR-derived assessment of rostral and caudal gene expression in MI-0 organoids. Forebrain, LHX2; midbrain, OTX2; hindbrain, HOXB4; cervical, HOXB5, cervical, HOXB8. 1/ ΔCt values are normalized to 18S rRNA expression levels, n = 3 batch-conversions per cell-line.

(E) Immunostaining of neuronal dendrites, axons, and cell bodies by MAP2, NF-H, and NEUN, respectively (scale bar, 25 μm).

(F) Immunostaining of $ChAT^+$ motor neurons (scale bar, 10 μm).

(G) Immunostaining of ChAT and parvalbumin (PV) shows differentiation of distinct neuronal subtypes (scale bar, 25 μm).

See also Figure S1.

(Zonouzi et al., 2019) (Figures 2H, 2I, and S2C). In order to characterize the spatial distribution of myelin at MI-12, sections from multiple organoids corresponding to superficial and deep areas of each organoid were stained with MBP. Distribution analysis of MBP^+ pixels revealed that myelin was predominantly found in the peripheral areas of each organoid, which coincided with dense orientated tracts of NF-H^+ axons (Figures S2C–S2G). A schematic representation of the distribution of myelin and its temporal development in this model is shown in Figure S2H.

Structural organization of myelinated axons and myelin compaction

Myelin thickness and the structural organization of myelinated axons into discrete subdomains can be used to assess the maturity of myelinated axons. To assess myelinated axon organization, we carried out immunostaining analyses of CASPR, CLAUDIN-11, ANKYRIN-G, as well as both NEUROFASCIN-155 (Nfasc155) and -186 (Nfasc186) localization using a pan-Neurofascin antibody. Figure 3A shows widespread accumulation of CASPR at the distal ends of myelin sheaths in MI-12 organoids and correct assembly and distribution of PNJs and nodes of Ranvier (Figure 3B) along distinct myelinated axons (Rasband and Peles, 2015). We next performed toluidine blue staining, which revealed widespread compact myelin in MI-12 organoids (Figure 3C). Using transmission electron microscopy, we confirmed the formation of compactly wrapped myelin lamellae in organoids derived from multiple cell lines (Figure 3D). Furthermore, g-ratio analysis of myelinated axons revealed comparable myelin thickness around axons in our organoids to that seen *in vivo* (mean g-ratio = 0.75 ± 0.09) (Figure 3E) (Hildebrand and Hahn, 1978). Therefore, oligodendrocytes in our organoids demonstrate compact myelin formation and myelinated axon organization, and thus, we refer to these myelinating organoids as myelinoids.

Nfasc155$^{-/-}$ patient-derived myelinoids recapitulate disease pathology of disordered myelinated axon organization

Having established a reliable system to study human myelination and myelinated axon organization, we next determined whether myelinoids generated from iPSCs (two independent clones) derived from a patient with a mutation in a major component of the PNJ would result in abnormal myelinated axon organization. A previously described nonsense mutation in the gene encoding Neurofascin (NFASC) cell adhesion molecules results in premature termination of the glial-expressed Nfasc155 product (Smigiel et al., 2018) (Figure S3A). The Nfasc155 protein is the glial component of the PNJ in myelinated fibers, and its loss in mice causes disruption of this adhesive junction (Sherman et al., 2005; Tait et al., 2000). *Nfasc155*$^{-/-}$ patient-derived myelinoids demonstrated comparable levels of neuronal and glial differentiation to control myelinoids. Patient-derived myelinoids, as expected, consisted of neurons ($ChAT^+$ and PV^+) and $GFAP^+$ astrocytes (Figures 4A–4C) and the proportion of oligodendroglial cells ($SOX10^+$/$DAPI^+$) as well as the proportion of mature oligodendroglial cells (MBP^+CNP^+/ $SOX10^+$) showed no differences to control myelinoids (Figures 4D–4F). Importantly, widespread myelin formation was evident in

CellPress
OPEN ACCESS

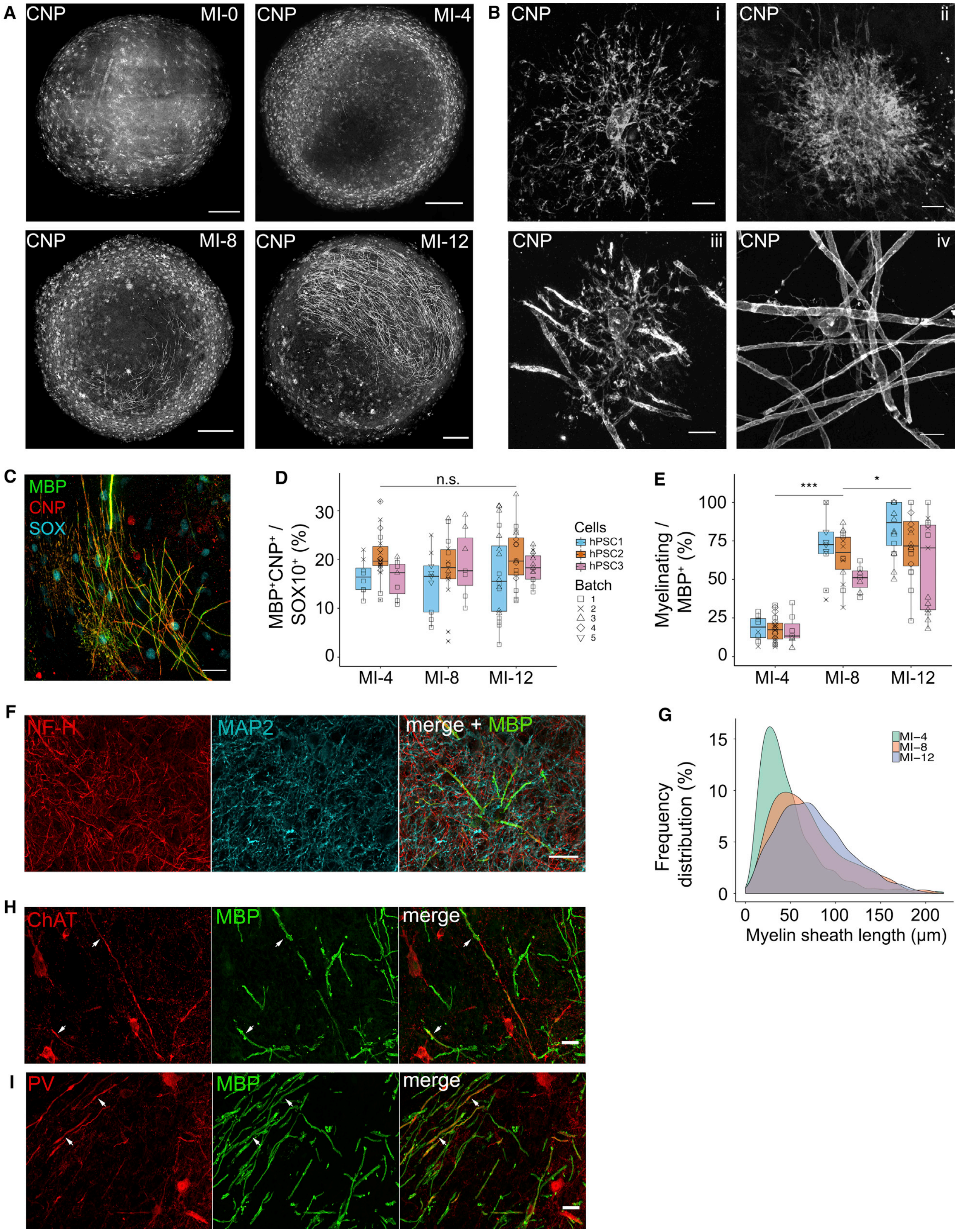

(legend on next page)

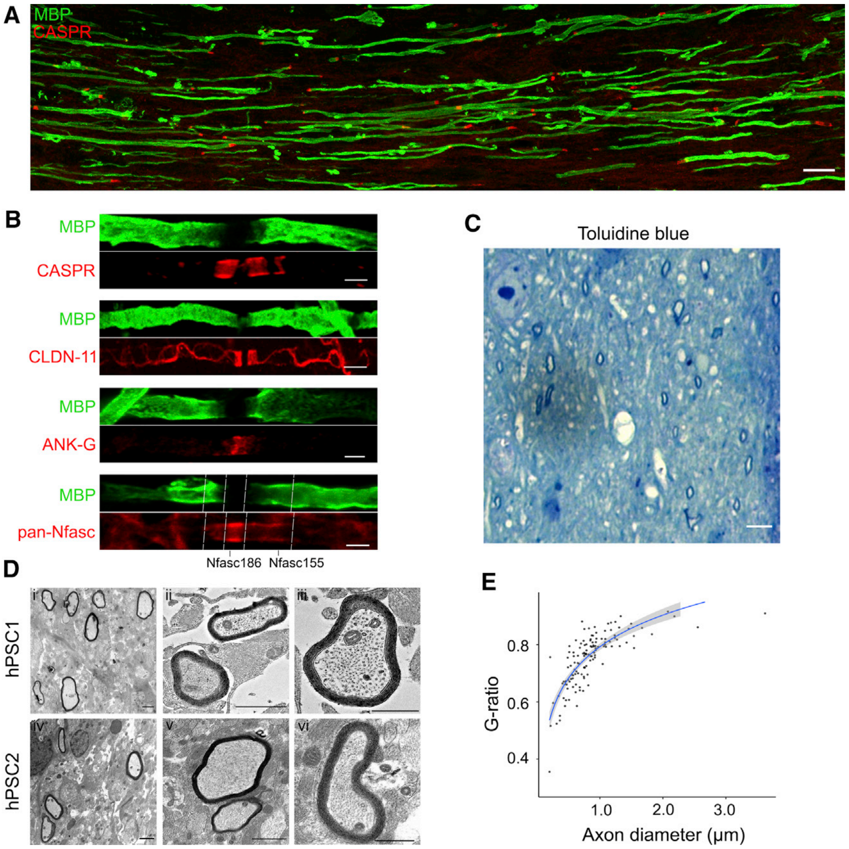

Figure 3. Structural organization of myelinated axons and myelin compaction

(A) Representative image of MBP and CASPR immunostaining at MI-12 (scale bar, 25 μm).

(B) Paranodal and nodal assembly in MI-12 myelinoids: CASPR and CLAUDIN-11 highlight PNJ formation and ANKYRIN-G highlights node formation. Pan-Nfasc identifies both Nfasc186 at the node and Nfasc155 at the paranode. White dotted lines mark these boundaries. Each pair of figures are from different myelinated axons. Scale bars, 5 μm (CASPR and CLDN-11) and 2 μm (ANK-G and pan-Nfasc).

(C) Toluidine blue staining of MI-12 myelinoids reveals presence of compact myelin (scale bar, 5 μm).

(D) Representative TEM images of compactly myelinated axons at MI-12 (scale bars for Di, Dii, Div, and Dv, 1 μm, scale bars for Diii and Dvi, 500 nm).

(E) Scatterplot of axon diameter against g-ratio (axon diameter divided by fiber diameter) with a logarithmic regression curve (124 axons from 6 myelinoids across distinct conversions from MI-8 onward).

Nfasc155$^{-/-}$ patient-derived myelinoids (Figure 4G) as found previously in mice (Sherman et al., 2005). To assess PNJ and node formation, myelinoids were stained with pan-Neurofascin as well as CASPR and ANKYRIN-G. While expression of Neurofascin at the node of Ranvier and ANKYRIN-G localization were comparable between *Nfasc155*$^{-/-}$ myelinoids and controls, we identified a lack of paranodal localization of both glial Neurofascin and its binding partner, CASPR (Figures 4H and 4I) across two independent clones of the *Nfasc155*$^{-/-}$ iPSC line (clone 1 and clone 2). These data show that iPSC myelinoids can be used to model disorders of myelinated axon organization.

Myelinoids predictably respond to pharmacological cues at both individual cell and whole-myelinoid levels

We next assessed whether human myelination could be pharmacologically manipulated in myelinoids and thus aimed to develop tools to quantify myelin changes at both the level of individual cells and globally across whole myelinoids. First, we traced individual myelin sheaths per cell to evaluate single oligodendrocyte morphology. Colocalization of CASPR at the distal ends of manually traced CNP$^+$ myelin sheaths provided confidence that we were accurately measuring individual myelin sheaths (Figures 5A and 5B). We observed on average 92% ± 4.8% of internodes at MI-12 had CASPR$^+$ PNJs (240 internodes across 6 myelinoids from 4 batch-conversions), demonstrating widespread paranodal organization. Under basal conditions and averaged across myelinoids derived from independent cell lines, we found that myelinating oligodendrocytes at MI-12 generated

Figure 2. Temporal development of myelin formation in iPSC myelinoids

(A) Representative images of myelin development between MI-0 and MI-12 (scale bar, 250 μm).

(B) Representative images of oligodendrocyte morphology between MI-0 to MI-4 (Bi and Bii) and MI-8 to MI-12 (Biii and Biv; scale bar, 10 μm).

(C) Representative image of SOX10$^+$CNP$^+$MBP$^+$ oligodendrocytes in MI-12 myelinoids (scale bar, 25 μm).

(D) The proportion of mature oligodendroglial cells (MBP$^+$CNP$^+$/SOX10$^+$) was stable between MI-4 and MI-12 at 18.1% (linear mixed effects regression, no change). Two images were taken per myelinoid from n = 22 (MI-4), 24 (MI-8), and 29 (MI-12) myelinoids across 3 cell lines (3–5 batch-conversions each).

(E) The proportion of mature MBP$^+$CNP$^+$ oligodendrocytes engaged in myelination increased by 4-fold between MI-4 and MI-12 (95% CI: 3.42- to 4.89-fold; $p < 0.001$); generalized linear mixed model (GLMM) with time point as fixed effect and unique myelinoid ID as random effect. Two images were taken per myelinoid from n = 22 (MI-4), 24 (MI-8), and 29 (MI-12) myelinoids across 3 cell lines (3–5 batch-conversions each).

(F) Immunostaining of MBP, NF-H, and MAP2 shows colocalization of myelin only on NF-H$^+$ axons (scale bar, 25 μm).

(G) Frequency distribution of MBP$^+$ myelin sheath lengths between MI-4 and MI-12. Myelin sheath length increased by 52% between MI-4 and MI-12 (95% CI: 44% to 60%; $p < 0.001$); GLMM with time point as fixed effect and unique myelinoid ID as random effect, n = 1,061 sheaths from 6 myelinoids (MI-4), 754 sheaths from 5 myelinoids (MI-8), 1,083 sheaths from 5 myelinoids (MI-12).

(H) Representative images of myelinated ChAT$^+$ axons (white arrows), scale bar, 10 μm.

(I) Representative images of myelinated PV$^+$ axons (white arrows), scale bar, 10 μm.

Boxplots show the medians, interquartile ranges and Tukey-style whiskers that extend to 1.5 times the interquartile range. n.s. = not significant, *$p < 0.05$, **$p < 0.01$, ***$p < 0.001$. See also Figure S2.

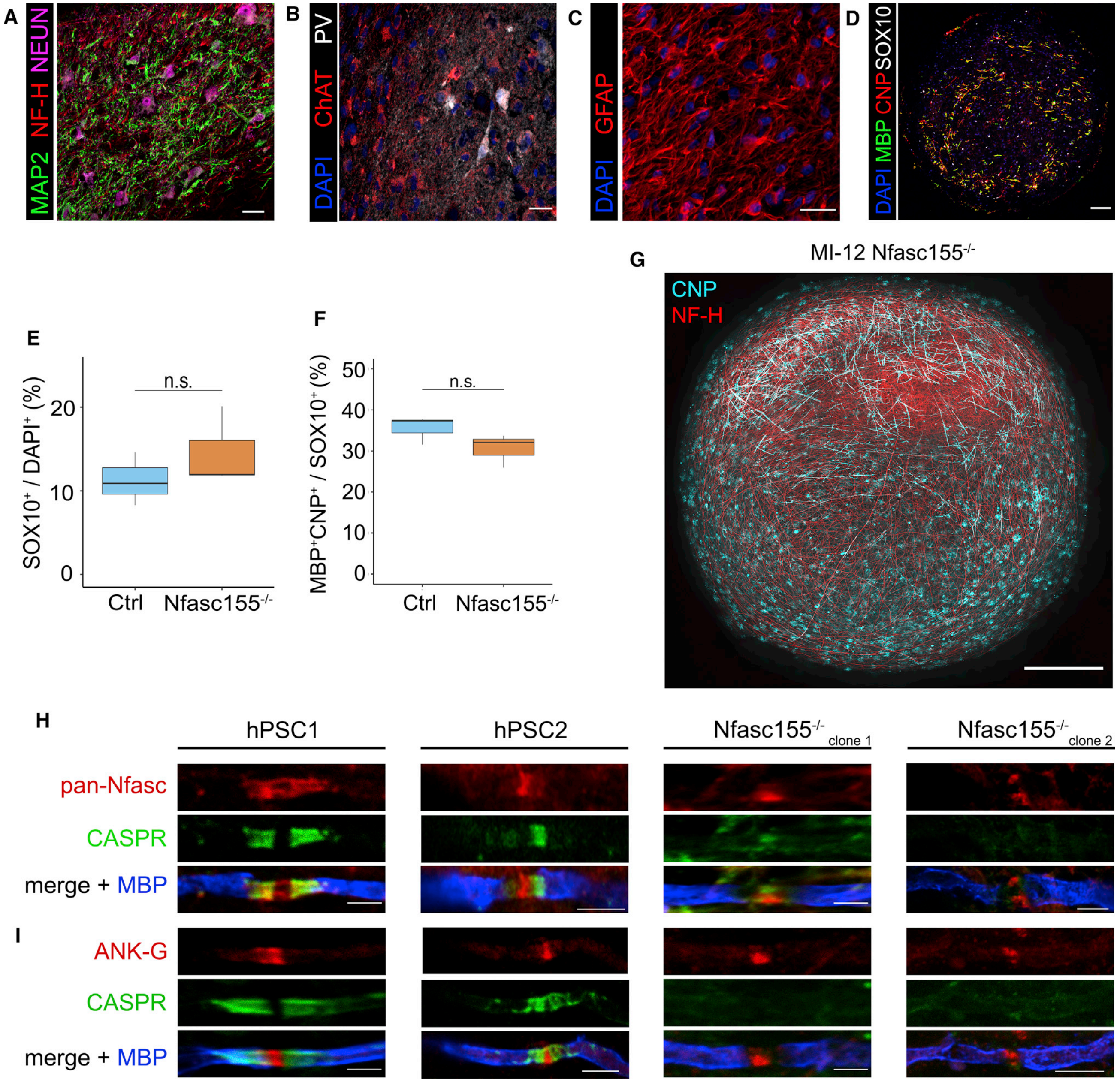

Figure 4. *Nfasc155*$^{-/-}$ patient-derived myelinoids recapitulate disease pathology of disordered myelinated axon organization

(A) Immuno-staining of neuronal dendrites, axons, and cell bodies in *Nfasc155*$^{-/-}$ MI-12 myelinoids by MAP2, NF-H, and NEUN respectively (scale bar, 25 μm).
(B) Immunostaining of ChAT^{+} and PV^{+} neurons in *Nfasc155*$^{-/-}$ MI-12 myelinoids (scale bar, 25 μm).
(C) Immunostaining of DAPI and GFAP^{+} astrocytes in *Nfasc155*$^{-/-}$ MI-12 myelinoids (scale bar, 25 μm).
(D) Representative image of cryosectioned *Nfasc155*$^{-/-}$ MI-12 myelinoid stained with DAPI, MBP, CNP, and SOX10 (scale bar, 25 μm).
(E) Analysis of the proportion of SOX10^{+} oligodendroglial cells showed no difference between Ctrl and *Nfasc155*$^{-/-}$ MI-12 myelinoid cryosections (GLMM with cell-line as fixed effect and unique myelinoid ID as random effect); n = 3 myelinoids each.
(F) Analysis of the proportion of mature MBP^{+} oligodendroglial cells showed no difference between Ctrl and *Nfasc155*$^{-/-}$ MI-12 myelinoid cryosections (GLMM with cell-line as fixed effect and unique myelinoid ID as random effect, n = 3 myelinoids each.
(G) Representative image of *Nfasc155*$^{-/-}$ patient-derived myelinoid at MI-12 (scale bar, 250 μm).
(H) Representative images of pan-Nfasc, CASPR and MBP across two Ctrl cell lines and two independently generated *Nfasc155*$^{-/-}$ cell-lines (clone 1 and clone 2) at MI-12. *Nfasc155*$^{-/-}$ myelinoids lack paranodal neurofascin expression and demonstrate disrupted PNJ formation. Axonally expressed neurofascin at the node remains intact (5–10 nodes analyzed per condition from < 3 myelinoids each; scale bar, 2 μm).
(I) Representative images of ankyrin-G, CASPR and MBP across two Ctrl cell-lines and two independently generated *Nfasc155*$^{-/-}$ cell-lines at MI-12. ANK-G expression further demonstrates that nodal assembly is preserved in *Nfasc155*$^{-/-}$ myelinoids (5–10 nodes analyzed per condition from < 3 myelinoids each; scale bar, 2 μm).
Boxplots show the medians, interquartile ranges and Tukey-style whiskers that extend to 1.5 times the interquartile range. n.s. = not significant. See also Figure S3.

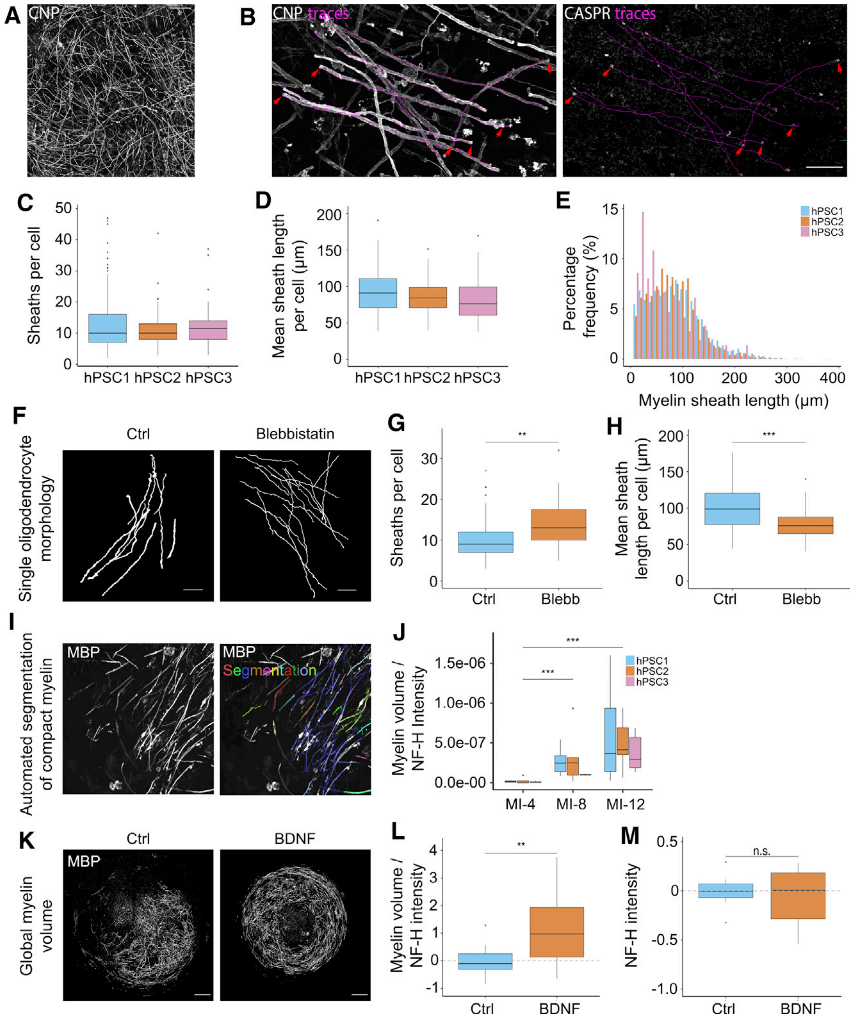

Figure 5. Myelinoids predictably respond to pharmacological cues at both individual cell and whole-myelinoid levels

(A) Representative image of tiled area for tracing individual myelin sheaths per cell (scale bar, 100 μm).

(B) Manual tracing of CNP$^+$ myelin sheath lengths. CASPR$^+$ PNJs (red arrows) precisely overlap the distal ends of manually traced myelin sheath lengths (magenta) (scale bar, 100 μm).

(C) Analysis of sheath number per cell (hPSC1: mean 12.5 ± 8.5 sheaths, hPSC2: 11.3 ± 5.1 sheaths, hPSC3: 12.3 ± 7.3 sheaths). n = 211 cells across 17 myelinoids (hPSC1), 110 cells across 9 myelinoids (hPSC2), and 90 cells across 9 myelinoids (hPSC3) from 2–5 conversions each.

(D) Analysis of mean sheath length per cell (hPSC1: mean 93.0 μm ± 29.8 μm, hPSC2: 85.6 μm ± 20.4 μm, hPSC3: 67.5 μm ± 28.5 μm). n = 211 cells across 17 myelinoids (hPSC1), 110 cells across 9 myelinoids (hPSC2), and 90 cells across 9 myelinoids (hPSC3) from 2–5 conversions each.

(E) Percentage frequency distribution of myelin sheath lengths at MI-12. n = 211 cells across 17 myelinoids (hPSC1), 110 cells across 9 myelinoids (hPSC2), and 90 cells across 9 myelinoids (hPSC3) from 2–5 conversions each.

(F) Skeletonized CNP$^+$ myelin sheaths of individual cells from Ctrl and blebbistatin-treated cultures (scale bar, 25 μm).

(G) Sheath number per cell was increased in blebbistatin-treated cultures by 44.5% compared to Ctrl (95% CI: 10% to 89%; p = 0.007); GLMM with treatment as fixed effect and unique myelinoid ID and batch-conversion as random effects; n = 94 cells from 7 myelinoids (Ctrl) and 60 cells from 6 myelinoids (blebb) across 2 conversions.

(H) Mean sheath length per cell was decreased in blebbistatin-treated cultures by 24% compared to Ctrl (95% CI: 10% to 35%; p < 0.001); GLMM with treatment as fixed effect and unique myelinoid ID and batch-conversion as random effects; n = 94 cells from 7 myelinoids (Ctrl) and 60 cells from 6 myelinoids (blebb) across 2 conversions.

(I) Representative images demonstrating automated segmentation of MBP$^+$ myelin sheaths at a particular Z-step. MBP expression is shown in greyscale and segmented objects overlaid in color.

(J) Automated analysis of myelin volume normalized to NF-H intensity over time revealed an overall 8-fold increase between MI-4 and MI-8 (95% CI: 3- to 24-fold; p = 0.006) and a 15-fold increase between MI-4 and MI-12 (95% CI: 6- to 40-fold; p < 0.0001); GLMM with timepoints as fixed effects and batch-conversion and cell-lines as random effects. n = 19 (MI-4), 13 (MI-8) and 34 (MI-12) myelinoids across three cell-lines (2–7 conversions each).

(K) Representative images of MBP expression in Ctrl and BDNF-treated whole-mounted myelinoids (scale bar, 250 μm).

(L) Automated analysis of myelin volume normalized to NF-H intensity revealed a 2.09-fold increase in BDNF-treated myelinoids (95% CI: 1.13-fold to 2.74-fold; p = 0.0024); GLMM with treatment as fixed effect and batch-conversion as random effect). n = 13 (Ctrl) and 13 (BDNF) myelinoids from 3 conversions each.

(M) Automated analysis of NF-H intensity showed no change between Ctrl and BDNF-treated myelinoids (GLMM with treatment as fixed effect and batch-conversion as random effect). n = 13 (Ctrl) and 13 (BDNF) myelinoids from 3 conversions each.

Boxplots show the medians, interquartile ranges and Tukey-style whiskers that extend to 1.5 times the interquartile range. n.s. = not significant, ***p* < 0.01, ****p* < 0.001. See also Figure S4.

a mean of 13 ± 8 sheaths per cell with a mean sheath length per cell of 87 μm ± 27.9 μm (Figures 5C and 5D). A frequency distribution curve of myelin sheath lengths is shown in Figure 5E. Myelinoids treated with blebbistatin, a non-muscle myosin II inhibitor and known modulator of myelin structure (Wang et al., 2008), between MI-0 and MI-12 exhibited a 44.5% increase in myelin sheath number per cell (Figures 5F and 5G). Mean sheath length per cell was reduced by 24% (Figure 5H). These findings match those found in studies of rodent oligodendrocytes (Wang et al., 2008) and show that the myelinating profile of individual oligodendrocytes can be modulated pharmacologically. In order to measure global levels of myelination, we developed an automated method of image acquisition and analysis using an ImageXpress microconfocal with MetaXpress software that permitted unbiased 3D quantification of myelin volume and axonal density (Figures 5I and 5J). Selective segmentation of myelinated axons is demonstrated in Figure 5J. We observed that myelin volume was closely correlated with axonal density (assessed by quantifying NF-H$^+$ intensity) (R^2 = 0.6, Figure S4) as has been shown previously (Almeida et al., 2011; Lundgaard

CellPress
OPEN ACCESS

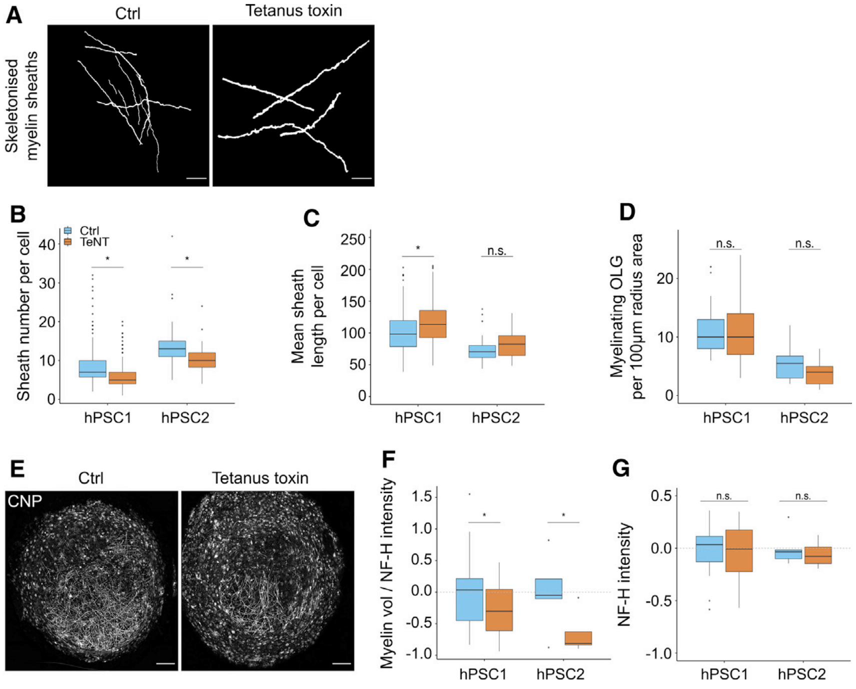

Figure 6. TeNT suppresses human myelination

(A) Skeletonised CNP+ myelin sheaths of individual cells from Ctrl and TeNT-treated cultures (scale bar, 25 μm).

(B) Sheath number per cell in Ctrl and TeNT-treated myelinoids. There was an overall 20% reduction in sheath number per cell in TeNT-treated myelinoids (95% CI: 6% to 31%; $p = 0.006$); GLMM with treatment as fixed effect and unique myelinoid ID and cell-line as random effects. hPSC1: 19% reduction (95% CI: 3% to 33%; $p = 0.02$); hPSC2: 23% reduction (95% CI: 0.4% to 40%; $p = 0.04$); n = 314 cells from 23 myelinoids (Ctrl) and 290 cells from 20 myelinoids (TeNT) across two cell-lines and 5 conversions.

(C) Mean sheath length per cell in Ctrl and TeNT-treated myelinoids. There was an overall 14% increase in mean sheath length per cell in TeNT-treated myelinoids (95% CI: 4% to 21%; $p < 0.01$); GLMM with treatment as fixed effect and unique myelinoid ID and cell-line as random effects). hPSC1: 12.5% increase (95% CI: 2% to 23%; $p = 0.018$); hPSC2: no statistical difference; n = 314 cells from 23 myelinoids (Ctrl) and 290 cells from 20 myelinoids (TeNT) across two cell-lines and 5 conversions.

(D) Nearest neighbor analysis shows no change in oligodendrocyte density between Ctrl and TeNT-treated myelinoids (GLMM with treatment as fixed effect and unique myelinoid ID and cell-line as random effects). n = 49 cells from 12 myelinoids (Ctrl) and 51 cells from 9 myelinoids (TeNT) across two cell-lines and 4 conversions.

(E) Representative images of Ctrl and TeNT-treated myelinoids (scale bar, 250 μm).

(F) Automated analysis of myelin volume normalized to NF-H intensity demonstrated an overall reduction of 38% in TeNT-treated myelinoids (95% CI: 4% to 52%; $p = 0.017$); GLMM with treatment as fixed effect and individual batch-conversions and cell-lines as random effects). hPSC1: 35% reduction (95% CI: 1.4% to 57% $p = 0.043$); hPSC2: 65% reduction (95% CI: 4.1% to 87.3%; $p = 0.041$); n = 29 (Ctrl) and 22 (TeNT) myelinoids across two cell lines and 5 conversions.

(G) Automated analysis of NF-H intensity (GLMM with treatment as fixed effect and individual batch-conversions and cell-lines as random effects, no change. n = 29 (Ctrl) and 22 (TeNT) myelinoids across two cell lines and 5 conversions.

Boxplots show the medians, interquartile ranges and Tukey-style whiskers that extend to 1.5 times the interquartile range. n.s. = not significant, $^{*}p < 0.05$. See also Figure S5.

et al., 2013). Therefore, to standardize the analysis of myelin volume across myelinoids, we normalized it to the integrative NF-H intensity measure for each myelinoid. Temporal analysis of myelin volume—normalized to NF-H intensity—demonstrated significantly increased myelin volume over time (Figure 5J). To potentiate myelin volume, myelinoids were treated with BDNF, which has previously been shown to enhance myelin formation (Lundgaard et al., 2013; Xiao et al., 2010). BDNF-treated myelinoids demonstrated a 2-fold increase in myelin volume—normalized to NF-H intensity—without affecting NF-H intensity (Figures 5K–5M), indicating that widespread effects on human myelination can be assessed using our model.

Tetanus toxin (TeNT) suppresses human myelination

Numerous cellular and animal models have shown that oligodendrocytes modulate myelin structure in response to changes in neuronal activity (Gibson et al., 2014; Hines et al., 2015; Mensch et al., 2015; Mitew et al., 2018; Wake et al., 2011). No evidence, to date, has been reported of changes to human myelination per se in response to altered neuronal activity. To address this, we sought to assess whether *in vitro* human myelination could be suppressed by blocking synaptic vesicle (SV) release. Tetanus toxin (TeNT) blocks SV release via proteolytic cleavage of synaptobrevin 1/2 (VAMP2), thereby suppressing both spontaneous as well as action potential-evoked SV release (Kim et al., 1984), including in stem cell-derived neurons (Beske et al., 2016). TeNT suppresses myelin formation both *in vitro* and *in vivo* in model systems (Hines et al., 2015; Mensch et al., 2015; Wake et al., 2011) and has been shown to reduce the number of myelin sheaths made by individual oligodendrocytes in the developing spinal cord of zebrafish, via a non-cell-autonomous mechanism (Mensch et al., 2015). To examine the impact of TeNT on human myelination, myelinoids were chronically treated with TeNT for 12 weeks at a concentration previously shown to suppress SV exocytosis (3nM; Morton et al., 2015). We found that TeNT-treated myelinoids, derived from two independent cell lines, showed a 20% reduction in the number of sheaths made by individual oligodendrocytes (Figures 6A and 6B). Concomitantly, we observed an increase in mean sheath length per cell (Figure 6C). A nearest neighbor analysis (Figure S5A) was performed to determine CNP+ oligodendrocyte density, which was found to be unchanged between Ctrl and TeNT-treated myelinoids (Figure 6D). To determine whether TeNT might directly affect oligodendrocyte morphology, noting that oligodendrocytes express SNARE proteins and that VAMP2 disruption alters oligodendrocyte morphogenesis (Sloane and Vartanian, 2007),

Table 1. Comparisons between different myelinating organoid protocols

		Madhavan et al., 2018	Marton et al., 2019	Kim et al., 2019	This study
Features of myelin	MBP^+ oligodendrocytes	+	+	+	+
	Widespread axonal ensheathment	–	–	–	+
	Widespread myelin compaction	–	–	–	+
	Myelinated axon organization	–	–	–	+
Technical differences	regionalization	cortical	cortical	cortical	Spinal cord
	reported culture time until appearance of mature oligodendrocytes	84 days	100 days	84 days	59 days
	culture method	ultra-low attachment dish + Geltrex	ultra-low attachment dish	ultra-low attachment dish	ultra-low attachment dish + cell culture insert
Pros/cons	study of human myelin development	+	+	+	++
	reduced culture time	+	+	+	++
	application in neuropsychiatric conditions	+	+	+	±

dissociated MI-0 spheroids were maintained as a monolayer for 14 days in the presence or absence of TeNT (Figure S5B). Sholl analysis of MBP^+ oligodendrocytes at day 14 showed no difference in oligodendrocyte branching or total outgrowth between Ctrl and TeNT-treated cultures (Figures S5C and S5D), suggesting that TeNT-induced hypomyelination does not occur via a cell-autonomous mechanism. Finally, to determine whether reduction in sheath number per cell would result in a global reduction in myelination, we performed automated quantification of myelin volume in TeNT-treated myelinoids. We found a 38% reduction in myelin volume relative to NF-H intensity in TeNT-treated myelinoids (Figures 6E and 6F). NF-H intensity was not affected by TeNT treatment, indicating that the effect on myelination was not due to general disruption to axonal development (Figure 6G). Thus, reduced vesicular release in human myelinating cultures suppresses myelin development, providing an opportunity for in-depth cellular, molecular, and functional analyses of human adaptive myelination.

DISCUSSION

We describe a model of myelination that recapitulates the three-dimensional architecture of myelinated axons for investigation of human myelin biology. Recognition that oligodendrocytes and myelin have multiple roles beyond saltatory conduction and are pivotal players in the dynamic cellular interplay of the CNS in health and disease underlines the importance of human models of oligodendrocyte-neuron interaction. This is emphasized by the growing number of conditions across the life course where dysregulation or loss of myelin is believed to play an important role (Fields and Bukalo, 2020; Mot et al., 2018; Nave and Ehrenreich, 2014).

Human stem cell-derived forebrain-patterned organoid models have previously shown oligodendrocyte maturation through myelination (Madhavan et al., 2018; Marton et al., 2019; Kim et al., 2019), but such systems lack widespread compact myelination and evidence of myelinated axon organization into functional domains. We hypothesized that the limited myelination and absence of paranodal and nodal structures in these studies may reflect their rostral regional identity and the later developmental acquisition of myelin in the brain compared with caudal structures (Back et al., 2002; Jakovcevski et al., 2009). Accordingly, we generated ventral spinal cord-patterned organoids to mimic the comparatively accelerated development of spinal cord myelination (Weidenheim et al., 1993). Table 1 summarizes key differences between published protocols. It would thus be of considerable interest to evaluate whether forebrain-patterned organoid preparations develop features of more complete myelination with prolonged culture. Regionally distinct human myelinating systems provide an opportunity to investigate cortical and spinal cord oligodendrocyte functional heterogeneity as well as model myelin diseases that show anatomical variation in predilection (Table 1). The use of cell culture inserts that maintain organoids at an air-liquid interface is also of interest (Table 1). These conditions provide enhanced oxygen availability and tissue survival (Klasvogt et al., 2017). Indeed, cerebral organoids cultured at an air-liquid interface demonstrate improved survival and outgrowth of neurons (Giandomenico et al., 2019). Increased oxygenation may also facilitate enhanced metabolic support to OPCs and pre-myelinating oligodendrocytes as the shift from oxidative phosphorylation to glycolysis does not occur until after developmental myelination is complete (Fünfschilling et al., 2012; Rao et al., 2017). While our study establishes a reproducible platform for the evaluation of human myelin biology, we also note technical considerations for future development and application of the model. A degree of biological variability between cell lines and batches was seen in our data, which can be explicitly modeled as random effects, as was performed in this study. Our integrative intensity measures of NF-H staining could also be further evolved in future work by adopting analytic methods to account for variation in $NF\text{-}H^+$ tract area and density between myelinoids.

The ability to recapitulate compact myelin formation and myelinated axon organization allows the study of nodal assembly and disruption in health and disease. Here, we demonstrated that patient-derived myelinoids recapitulated the key pathology

CellPress
OPEN ACCESS

of Nfasc155 deficiency, namely impaired paranode formation. Importantly, given that *Nfasc155*$^{-/-}$ mice prematurely die between P17–P20, investigations into the long-term impact of Nfasc155 deficiency or other mutations on nodal domain stability and axonal health may be well served using iPSC myelinoids (Pillai et al., 2009; Roche et al., 2014). Furthermore, using an automated platform of image analysis, the ability to screen for myelin-related phenotypes across patient-derived myelinoids as well as pharmacological rescue of myelin phenotypes is a further application of this model. Several studies have revealed key pathways and compounds that promote oligodendrocyte differentiation and myelination (Deshmukh et al., 2013; Early et al., 2018; Hubler et al., 2018; Mei et al., 2014, 2016). With further adaptation, such as miniaturization into multi-well plate format and the use of myelin-specific fluorescent markers, screening for small molecules that promote human myelination in iPSC myelinoids represents an important goal.

Multiple lines of accumulating evidence dating back to *in vivo* observations in dark reared mice through to optogenetic or DREADD-based modulation provide a strong evidence base for neuronal activity-regulated myelination (Gibson et al., 2014; Gyllensten and Malmfors, 1963; Mitew et al., 2018). However, whether human myelinating oligodendrocytes are capable of responding to changes in neuronal activity has not been possible to directly study. To begin to address the effect of neuronal activity on myelination, we used TeNT to block SV release. Blocking SV release has been shown to lead to preferential myelin targeting to active axons and a reduction in internodes per oligodendrocyte both *in vitro* and *in vivo* (Hines et al., 2015; Koudelka et al., 2016; Mensch et al., 2015; Wake et al., 2011). We demonstrated that myelinoids exposed to TeNT showed a reduction in both internode number per oligodendrocyte and a reduction in global myelin volume that mirrors data obtained from analogous *in vivo* experiments of TeNT-expressing zebrafish (Mensch et al., 2015). Oligodendrocyte density was unchanged following TeNT exposure in myelinoids, in line with the minimal effect observed in zebrafish. Furthermore, we observed differences in the degree by which TeNT affected sheaths per cell and global myelin volume (20% versus 38% reduction, respectively) and between the two cell lines. Noting that automated segmentation is an intensity-based analysis, these differences may be explained by the capture of additional myelin sheath dimensions including myelin sheath thickness. Furthermore, as the hypomyelinating effect of TeNT is dependent on the level of intrinsic neuronal activity in myelinoid cultures, variation in basal activity between batches of myelinoids may add to the differences observed. To comprehensively interrogate the influence of activity on myelination, it would thus be important to examine the effect of systematic modulation and bidirectional tuning of neuronal activity using a variety of approaches from cell type-specific expression of chemogenetic tools to pharmacological manipulation.

Collectively, our demonstration of a robust model of widespread compact myelination with appropriate nodal architecture establishes a tractable and quantifiable platform for evaluation of human myelin biology in a tissue-level context. The ability to assess oligodendrocyte cell biology, cell-cell interactions and disruption in disease is possible, with the potential for phenotypic screening of myelin development and pharmacological interrogation of developmental and activity-dependent processes.

Limitations of the study

As mentioned above, one limitation is that inferences made on myelin development in this model may not be generalizable due to the spinal cord identity of myelinoid cultures. As such, their use in studying myelin development in the context of certain neurological conditions may be limited. Second, given the extended time frame over which myelination occurs in this system, it can be challenging to optimize the dosing of small molecules/treatments that influence myelin development or neuronal activity. This could be improved by the use of cell type-specific reporters and optogenetic applications. Furthermore, a common limitation of organoid models is that they lack the full complement and diversity of cell types found *in vivo*. Microglia are absent in these organoids and yet play critical roles in neurodevelopment (Hagemeyer et al., 2017; Li and Barres, 2018). Several methods to incorporate microglia into developing organoids have been successful including patterning of intrinsic cells (Ormel et al., 2018) or transplantation of exogenously derived cells (Abud et al., 2017; Banerjee et al., 2020). Application of such methods with iPSC myelinoids would provide an opportunity to study the role of microglia in myelin development, degradation, and repair, the role of which remains largely unknown.

STAR★METHODS

Detailed methods are provided in the online version of this paper and include the following:

- KEY RESOURCES TABLE
- RESOURCE AVAILABILITY
  - Lead contact
  - Materials availability
  - Data and code availability
- EXPERIMENTAL MODEL AND SUBJECT DETAILS
  - Culture of human iPSCs
- METHOD DETAILS
  - Generation of iPSC myelinoids
  - Dissociated myelinoid cultures
  - Immunostaining of whole-mounted myelinoids
  - Cryopreservation and immuno-staining
  - Image acquisition
  - Transmission electron microscopy
  - PCR amplification of Nfasc155
  - Quantitative RT-PCR
- QUANTIFICATION AND STATISTICAL ANALYSIS
  - Image analysis
  - Automated analysis of myelin volume
  - Statistical analysis

SUPPLEMENTAL INFORMATION

Supplemental information can be found online at https://doi.org/10.1016/j.devcel.2021.04.006.

ACKNOWLEDGMENTS

O.G.J. was funded by the Euan MacDonald Centre for Motor Neuron Disease Research and the College of Medicine and Veterinary Medicine, University of Edinburgh. B.T.S. is funded by Rowling/DRI fellowship. S.K.B. received support from the National Health and Medical Research Council and Australian Research Council (APP1110040). P.J.B. was supported by an award from the Wellcome Trust (grant no. 107008). D.A.L. was supported by Wellcome Trust Senior research fellowships (102836/Z/13/Z and 214244/Z/18/Z). The Chandran laboratory is funded by a Centre of Excellence award by the MS Society UK, an MRC stem cell partnership award, an MRC Dementias Platform UK award and the UK Dementia Research Institute, which receives its funding from UK DRI Ltd., funded by the UK Medical Research Council, Alzheimer's Society, and Alzheimer's Research UK. We thank Joanna Brown and Steve Mitchell for assistance in preparing electron microscopy samples and Nicola Miller, Karen Gladstone, Jyoti Nanda, and James Cooper for assistance in iPSC culturing and maintenance. The graphical abstract for this manuscript was created using Biorender.com.

AUTHOR CONTRIBUTIONS

O.G.J., B.T.S., C.ff.-C., and S.C. conceived and designed the study. Material preparation and data collection were performed by O.G.J., K.B., D.W.H., and D.S. Analysis and interpretation were performed by O.G.J., B.T.S., D.M., K.B., P.C., S.K.B., N.A.V., P.J.B., C.ff.-C., D.A.L., and S.C.. R.S. is the neurologist managing the *Nfasc*$^{-/-}$ patient and who obtained the fibroblasts. R.P. is the geneticist who identified the nonsense mutation. P.J.B. confirmed that the nonsense mutation identified in the patient causes the selective loss of the Nfasc155 protein. O.G.J., B.T.S., D.A.L., and S.C. wrote the manuscript. All authors approved the manuscript.

DECLARATION OF INTERESTS

S.C. is a co-founder of Pheno Therapeutics. All other authors declare no competing interests.

Received: July 14, 2020
Revised: January 20, 2021
Accepted: April 6, 2021
Published: May 3, 2021

REFERENCES

Aarts, E., Verhage, M., Veenvliet, J.V., Dolan, C.V., and van der Sluis, S. (2014). A solution to dependency: using multilevel analysis to accommodate nested data. Nat. Neurosci. *17*, 491–496.

Abud, E.M., Ramirez, R.N., Martinez, E.S., Healy, L.M., Nguyen, C.H.H., Newman, S.A., Yeromin, A.V., Scarfone, V.M., Marsh, S.E., Fimbres, C., et al. (2017). iPSC-derived human microglia-like cells to study neurological diseases. Neuron *94*, 278–293.e9.

Almeida, R.G., Czopka, T., Ffrench-Constant, C., and Lyons, D.A. (2011). Individual axons regulate the myelinating potential of single oligodendrocytes in vivo. Development *138*, 4443–4450.

Alvarez, F.J., Jonas, P.C., Sapir, T., Hartley, R., Berrocal, M.C., Geiman, E.J., Todd, A.J., and Goulding, M. (2005). Postnatal phenotype and localization of spinal cord V1 derived interneurons. J. Comp. Neurol. *493*, 177–192.

Ameis, S.H., and Catani, M. (2015). Altered white matter connectivity as a neural substrate for social impairment in autism spectrum disorder. Cortex *62*, 158–181.

Back, S.A. (2017). White matter injury in the preterm infant: pathology and mechanisms. Acta Neuropathol *134*, 331–349.

Back, S.A., Luo, N.L., Borenstein, N.S., Volpe, J.J., and Kinney, H.C. (2002). Arrested oligodendrocyte lineage progression during human cerebral white matter development: dissociation between the timing of progenitor differentiation and myelinogenesis. J. Neuropathol. Exp. Neurol. *61*, 197–211.

Banerjee, P., Paza, E., Perkins, E.M., James, O.G., Kenkhuis, B., Lloyd, A.F., Burr, K., Story, D., Yusuf, D., He, X., et al. (2020). Generation of pure monocultures of human microglia-like cells from induced pluripotent stem cells. Stem Cell Res *49*, 102046.

Bechler, M.E., Swire, M., and Ffrench-Constant, C. (2018). Intrinsic and adaptive myelination-A sequential mechanism for smart wiring in the brain. Dev. Neurobiol. *78*, 68–79.

Beske, P.H., Bradford, A.B., Grynovicki, J.O., Glotfelty, E.J., Hoffman, K.M., Hubbard, K.S., Tuznik, K.M., and McNutt, P.M. (2016). Botulinum and tetanus neurotoxin-induced blockade of synaptic transmission in networked cultures of human and rodent neurons. Toxicol. Sci. *149*, 503–515.

Bilican, B., Livesey, M.R., Haghi, G., Qiu, J., Burr, K., Siller, R., Hardingham, G.E., Wyllie, D.J.A., and Chandran, S. (2014). Physiological normoxia and absence of EGF is required for the long-term propagation of anterior neural precursors from human pluripotent cells. PLoS One *9*, e85932.

Brooks, M., Kristensen, K., Benthem, K., Magnusson, A., Berg, C., Nielsen, A., Skaug, H., Mächler, M., and Bolker, B. (2017). glmmTMB balances speed and flexibility Among packages for zero-inflated generalized linear mixed modeling. R J *9*, 378.

Castelijns, B., Baak, M.L., Timpanaro, I.S., Wiggers, C.R.M., Vermunt, M.W., Shang, P., Kondova, I., Geeven, G., Bianchi, V., de Laat, W., et al. (2020). Hominin-specific regulatory elements selectively emerged in oligodendrocytes and are disrupted in autism patients. Nat. Commun. *11*, 301.

Dasen, J.S., and Jessell, T.M. (2009). Chapter six hox networks and the origins of motor neuron diversity, H. Genes, ed. (Elsevier)), pp. 169–200.

Deshmukh, V.A., Tardif, V., Lyssiotis, C.A., Green, C.C., Kerman, B., Kim, H.J., Padmanabhan, K., Swoboda, J.G., Ahmad, I., Kondo, T., et al. (2013). A regenerative approach to the treatment of multiple sclerosis. Nature *502*, 327–332.

Early, J.J., Cole, K.L., Williamson, J.M., Swire, M., Kamadurai, H., Muskavitch, M., and Lyons, D.A. (2018). An automated high-resolution in vivo screen in zebrafish to identify chemical regulators of myelination. eLife *7*.

Ferreira, T.A., Blackman, A.V., Oyrer, J., Jayabal, S., Chung, A.J., Watt, A.J., Sjöström, P.J., and van Meyel, D.J. (2014). Neuronal morphometry directly from bitmap images. Nat. Methods *11*, 982–984.

Fields, R.D., and Bukalo, O. (2020). Myelin makes memories. Nat. Neurosci. *23*, 469–470.

Fünfschilling, U., Supplie, L.M., Mahad, D., Boretius, S., Saab, A.S., Edgar, J., Brinkmann, B.G., Kassmann, C.M., Tzvetanova, I.D., Möbius, W., et al. (2012). Glycolytic oligodendrocytes maintain myelin and long-term axonal integrity. Nature *485*, 517–521.

Giandomenico, S.L., Mierau, S.B., Gibbons, G.M., Wenger, L.M.D., Masullo, L., Sit, T., Sutcliffe, M., Boulanger, J., Tripodi, M., Derivery, E., et al. (2019). Cerebral organoids at the air-liquid interface generate diverse nerve tracts with functional output. Nat. Neurosci. *22*, 669–679.

Gibson, E.M., Purger, D., Mount, C.W., Goldstein, A.K., Lin, G.L., Wood, L.S., Inema, I., Miller, S.E., Bieri, G., Zuchero, J.B., et al. (2014). Neuronal activity promotes oligodendrogenesis and adaptive myelination in the mammalian brain. Science *344*, 1252304.

Gyllensten, L., and Malmfors, T. (1963). Myelinization of the optic nerve and its dependence on visual function–a quantitative investigation in mice. J. Embryol. Exp. Morphol. *11*, 255–266.

Hagemeyer, N., Hanft, K.M., Akriditou, M.A., Unger, N., Park, E.S., Stanley, E.R., Staszewski, O., Dimou, L., and Prinz, M. (2017). Microglia contribute to normal myelinogenesis and to oligodendrocyte progenitor maintenance during adulthood. Acta Neuropathol *134*, 441–458.

Hildebrand, C., and Hahn, R. (1978). Relation between myelin sheath thickness and axon size in spinal cord white matter of some vertebrate species. J. Neurol. Sci. *38*, 421–434.

Hines, J.H., Ravanelli, A.M., Schwindt, R., Scott, E.K., and Appel, B. (2015). Neuronal activity biases axon selection for myelination in vivo. Nat. Neurosci. *18*, 683–689.

Hodge, R.D., Bakken, T.E., Miller, J.A., Smith, K.A., Barkan, E.R., Graybuck, L.T., Close, J.L., Long, B., Johansen, N., Penn, O., et al. (2019). Conserved cell types with divergent features in human versus mouse cortex. Nature *573*, 61–68.

Hossaini, M., Cardona Cano, S., van Dis, V., Haasdijk, E.D., Hoogenraad, C.C., Holstege, J.C., and Jaarsma, D. (2011). Spinal inhibitory interneuron pathology follows motor neuron degeneration independent of glial mutant superoxide dismutase 1 expression in SOD1-ALS mice. J. Neuropathol. Exp. Neurol. *70*, 662–677.

Huang, B., Wei, W., Wang, G., Gaertig, M.A., Feng, Y., Wang, W., Li, X.J., and Li, S. (2015). Mutant huntingtin downregulates myelin regulatory factor-mediated myelin gene expression and affects mature oligodendrocytes. Neuron *85*, 1212–1226.

Hubler, Z., Allimuthu, D., Bederman, I., Elitt, M.S., Madhavan, M., Allan, K.C., Shick, H.E., Garrison, E., T Karl, M., Factor, D.C., et al. (2018). Accumulation of 8,9-unsaturated sterols drives oligodendrocyte formation and remyelination. Nature *560*, 372–376.

Jäkel, S., Agirre, E., Mendanha Falcão, A., van Bruggen, D., Lee, K.W., Knuesel, I., Malhotra, D., Ffrench-Constant, C., Williams, A., and Castelo-Branco, G. (2019). Altered human oligodendrocyte heterogeneity in multiple sclerosis. Nature *566*, 543–547.

Jakovcevski, I., Filipovic, R., Mo, Z., Rakic, S., and Zecevic, N. (2009). Oligodendrocyte development and the onset of myelination in the human fetal brain. Front. Neuroanat. *3*, 5.

Kang, S.H., Li, Y., Fukaya, M., Lorenzini, I., Cleveland, D.W., Ostrow, L.W., Rothstein, J.D., and Bergles, D.E. (2013). Degeneration and impaired regeneration of gray matter oligodendrocytes in amyotrophic lateral sclerosis. Nat. Neurosci. *16*, 571–579.

Kelly, S., Jahanshad, N., Zalesky, A., Kochunov, P., Agartz, I., Alloza, C., Andreassen, O.A., Arango, C., Banaj, N., Bouix, S., et al. (2018). Widespread white matter microstructural differences in schizophrenia across 4322 individuals: results from the ENIGMA Schizophrenia DTI working group. Mol. Psychiatry *23*, 1261–1269.

Kim, H., Xu, R., Padmashri, R., Dunaevsky, A., Liu, Y., Dreyfus, C.F., and Jiang, P. (2019). Pluripotent stem cell-derived cerebral organoids reveal human oligodendrogenesis with dorsal and ventral origins. Stem Cell Rep *12*, 890–905.

Kim, Y.I., Lømo, T., Lupa, M.T., and Thesleff, S. (1984). Miniature end-plate potentials in rat skeletal muscle poisoned with botulinum toxin. J. Physiol. *356*, 587–599.

Klasvogt, S., Zuschratter, W., Schmidt, A., Kröber, A., Vorwerk, S., Wolter, R., Isermann, B., Wimmers, K., Rothkötter, H.J., and Nossol, C. (2017). Air-liquid interface enhances oxidative phosphorylation in intestinal epithelial cell line IPEC-J2. Cell Death Discov *3*, 17001.

Koudelka, S., Voas, M.G., Almeida, R.G., Baraban, M., Soetaert, J., Meyer, M.P., Talbot, W.S., and Lyons, D.A. (2016). Individual neuronal subtypes exhibit diversity in CNS myelination mediated by synaptic vesicle release. Curr. Biol. *26*, 1447–1455.

Li, Q., and Barres, B.A. (2018). Microglia and macrophages in brain homeostasis and disease. Nat. Rev. Immunol. *18*, 225–242.

Livesey, M.R., Magnani, D., Cleary, E.M., Vasistha, N.A., James, O.T., Selvaraj, B.T., Burr, K., Story, D., Shaw, C.E., Kind, P.C., et al. (2016). Maturation and electrophysiological properties of human pluripotent stem cell-derived oligodendrocytes. Stem Cells *34*, 1040–1053.

Longair, M.H., Baker, D.A., and Armstrong, J.D. (2011). Simple Neurite Tracer: open source software for reconstruction, visualization and analysis of neuronal processes. Bioinformatics *27*, 2453–2454.

Lubetzki, C., Sol-Foulon, N., and Desmazières, A. (2020). Nodes of Ranvier during development and repair in the CNS. Nat. Rev. Neurol. *16*, 426–439.

Lundgaard, I., Luzhynskaya, A., Stockley, J.H., Wang, Z., Evans, K.A., Swire, M., Volbracht, K., Gautier, H.O.B., Franklin, R.J.M., Ffrench-Constant, Charles, et al. (2013). Neuregulin and BDNF induce a switch to NMDA receptor-dependent myelination by oligodendrocytes. PLoS Biol *11*, e1001743.

Madhavan, M., Nevin, Z.S., Shick, H.E., Garrison, E., Clarkson-Paredes, C., Karl, M., Clayton, B.L.L., Factor, D.C., Allan, K.C., Barbar, L., et al. (2018). Induction of myelinating oligodendrocytes in human cortical spheroids. Nat. Methods *15*, 700–706.

Magnani, D., Chandran, S., Wyllie, D.J.A., and Livesey, M.R. (2019). In vitro generation and electrophysiological characterization of OPCs and oligodendrocytes from human pluripotent stem cells. Methods Mol. Biol. *1936*, 65–77.

Marton, R.M., Miura, Y., Sloan, S.A., Li, Q., Revah, O., Levy, R.J., Huguenard, J.R., and Paşca, S.P. (2019). Differentiation and maturation of oligodendrocytes in human three-dimensional neural cultures. Nat. Neurosci. *22*, 484–491.

Mei, F., Fancy, S.P.J., Shen, Y.-A.A., Niu, J., Zhao, C., Presley, B., Miao, E., Lee, S., Mayoral, S.R., Redmond, S.A., et al. (2014). Micropillar arrays as a high-throughput screening platform for therapeutics in multiple sclerosis. Nat. Med. *20*, 954–960.

Mei, F., Mayoral, S.R., Nobuta, H., Wang, F., Desponts, C., Lorrain, D.S., Xiao, L., Green, A.J., Rowitch, D., Whistler, J., and Chan, J.R. (2016). Identification of the kappa-opioid receptor as a therapeutic target for oligodendrocyte remyelination. J. Neurosci. *36*, 7925–7935.

Mensch, S., Baraban, M., Almeida, R., Czopka, T., Ausborn, J., El Manira, A., and Lyons, D.A. (2015). Synaptic vesicle release regulates myelin sheath number of individual oligodendrocytes in vivo. Nat. Neurosci. *18*, 628–630.

Mitew, S., Gobius, I., Fenlon, L.R., McDougall, S.J., Hawkes, D., Xing, Y.L., Bujalka, H., Gundlach, A.L., Richards, L.J., Kilpatrick, T.J., et al. (2018). Pharmacogenetic stimulation of neuronal activity increases myelination in an axon-specific manner. Nat. Commun. *9*, 306.

Morton, A., Marland, J.R.K., and Cousin, M.A. (2015). Synaptic vesicle exocytosis and increased cytosolic calcium are both necessary but not sufficient for activity-dependent bulk endocytosis. J. Neurochem. *134*, 405–415.

Mot, A.I., Depp, C., and Nave, K.A. (2018). An emerging role of dysfunctional axon-oligodendrocyte coupling in neurodegenerative diseases. Dialogues Clin. Neurosci. *20*, 283–292.

Nasrabady, S.E., Rizvi, B., Goldman, J.E., and Brickman, A.M. (2018). White matter changes in Alzheimer's disease: a focus on myelin and oligodendrocytes. Acta Neuropathol. Commun. *6*, 22.

Nave, K.A., and Ehrenreich, H. (2014). Myelination and oligodendrocyte functions in psychiatric diseases. JAMA Psychiatry *71*, 582–584.

Nordström, U., Maier, E., Jessell, T.M., and Edlund, T. (2006). An early role for WNT signaling in specifying neural patterns of Cdx and Hox gene expression and motor neuron subtype identity. PLoS Biol *4*, e252.

Noseworthy, J.H., Lucchinetti, C., Rodriguez, M., and Weinshenker, B.G. (2000). Multiple sclerosis. N. Engl. J. Med. *343*, 938–952.

Ormel, P.R., Vieira de Sá, R., van Bodegraven, E.J., Karst, H., Harschnitz, O., Sneeboer, M.A.M., Johansen, L.E., van Dijk, R.E., Scheefhals, N., van Berlekom, A.B., et al. (2018). Microglia innately develop within cerebral organoids. Nat. Commun. *9*, 4167.

Philippidou, P., Walsh, C.M., Aubin, J., Jeannotte, L., and Dasen, J.S. (2012). Sustained Hox5 gene activity is required for respiratory motor neuron development. Nat. Neurosci. *15*, 1636–1644.

Pillai, A.M., Thaxton, C., Pribisko, A.L., Cheng, J.G., Dupree, J.L., and Bhat, M.A. (2009). Spatiotemporal ablation of myelinating glia-specific neurofascin (Nfasc NF155) in mice reveals gradual loss of paranodal axoglial junctions and concomitant disorganization of axonal domains. J. Neurosci. Res. *87*, 1773–1793.

Preibisch, S., Saalfeld, S., and Tomancak, P. (2009). Globally optimal stitching of tiled 3D microscopic image acquisitions. Bioinformatics *25*, 1463–1465.

Rao, V.T.S., Khan, D., Cui, Q.L., Fuh, S.C., Hossain, S., Almazan, G., Multhaup, G., Healy, L.M., Kennedy, T.E., and Antel, J.P. (2017). Distinct age and differentiation-state dependent metabolic profiles of oligodendrocytes under optimal and stress conditions. PLoS One *12*, e0182372.

Rasband, M.N., and Peles, E. (2015). The nodes of ranvier: molecular assembly and maintenance. Cold Spring Harb. Perspect. Biol. *8*, a020495.

Roche, S.L., Sherman, D.L., Dissanayake, K., Soucy, G., Desmazieres, A., Lamont, D.J., Peles, E., Julien, J.P., Wishart, T.M., Ribchester, R.R., et al. (2014). Loss of glial neurofascin155 delays developmental synapse elimination at the neuromuscular junction. J. Neurosci. *34*, 12904–12918.

Sampaio-Baptista, C., and Johansen-Berg, H. (2017). White matter plasticity in the adult brain. Neuron *96*, 1239–1251.

Seidl, A.H. (2014). Regulation of conduction time along axons. Neuroscience *276*, 126–134.

Sherman, D.L., Tait, S., Melrose, S., Johnson, R., Zonta, B., Court, F.A., Macklin, W.B., Meek, S., Smith, A.J.H., Cottrell, D.F., and Brophy, P.J. (2005). Neurofascins are required to establish axonal domains for saltatory conduction. Neuron *48*, 737–742.

Simons, M., and Nave, K.A. (2015). Oligodendrocytes: myelination and axonal support. Cold Spring Harb. Perspect. Biol. *8*, a020479.

Sloane, J.A., and Vartanian, T.K. (2007). Myosin va controls oligodendrocyte morphogenesis and myelination. J. Neurosci. *27*, 11366–11375.

Smigiel, R., Sherman, D.L., Rydzanicz, M., Walczak, A., Mikolajkow, D., Krolak-Olejnik, B., Kosinska, J., Gasperowicz, P., Biernacka, A., Stawinski, P., et al. (2018). Homozygous mutation in the neurofascin gene affecting the glial isoform of neurofascin causes severe neurodevelopment disorder with hypotonia, amimia and areflexia. Hum. Mol. Genet. *27*, 3669–3674.

Stoppini, L., Buchs, P.A., and Muller, D. (1991). A simple method for organotypic cultures of nervous tissue. J. Neurosci. Methods *37*, 173–182.

Susuki, K., Chang, K.J., Zollinger, D.R., Liu, Y., Ogawa, Y., Eshed-Eisenbach, Y., Dours-Zimmermann, M.T., Oses-Prieto, J.A., Burlingame, A.L., Seidenbecher, C.I., et al. (2013). Three mechanisms assemble central nervous system nodes of Ranvier. Neuron *78*, 469–482.

Tait, S., Gunn-Moore, F., Collinson, J.M., Huang, J., Lubetzki, C., Pedraza, L., Sherman, D.L., Colman, D.R., and Brophy, P.J. (2000). An oligodendrocyte cell adhesion molecule at the site of assembly of the paranodal axo-glial junction. J. Cell Biol. *150*, 657–666.

Vasistha, N.A., Johnstone, M., Barton, S.K., Mayerl, S.E., Thangaraj Selvaraj, B., Thomson, P.A., Dando, O., Grünewald, E., Alloza, C., Bastin, M.E., et al. (2019). Familial t(1;11) translocation is associated with disruption of white matter structural integrity and oligodendrocyte-myelin dysfunction. Mol. Psychiatry *24*, 1641–1654.

Wake, H., Lee, P.R., and Fields, R.D. (2011). Control of local protein synthesis and initial events in myelination by action potentials. Science *333*, 1647–1651.

Wang, H., Tewari, A., Einheber, S., Salzer, J.L., and Melendez-Vasquez, C.V. (2008). Myosin II has distinct functions in PNS and CNS myelin sheath formation. J. Cell Biol. *182*, 1171–1184.

Weidenheim, K.M., Epshteyn, I., Rashbaum, W.K., and Lyman, W.D. (1993). Neuroanatomical localization of myelin basic protein in the late first and early second trimester human foetal spinal cord and brainstem. J. Neurocytol. *22*, 507–516.

Westlye, L.T., Walhovd, K.B., Dale, A.M., Bjørnerud, A., Due-Tønnessen, P., Engvig, A., Grydeland, H., Tamnes, C.K., Ostby, Y., and Fjell, A.M. (2010). Life-span changes of the human brain white matter: diffusion tensor imaging (DTI) and volumetry. Cereb. Cortex *20*, 2055–2068.

Wiggins, R.C., Chongjie, G., Delaney, C., and Samorajski, T. (1988). Development of axonal-oligodendroglial relationships and junctions during myelination of the optic nerve. Int. J. Dev. Neurosci. *6*, 233–243.

Williamson, J.M., and Lyons, D.A. (2018). Myelin dynamics Throughout life: an ever-changing landscape? Front. Cell. Neurosci. *12*, 424.

Wolf, N.I., Ffrench-Constant, C., and van der Knaap, M.S. (2021). Hypomyelinating leukodystrophies - unravelling myelin biology. Nat. Rev. Neurol. *17*, 88–103.

Xiao, J., Wong, A.W., Willingham, M.M., van den Buuse, M., Kilpatrick, T.J., and Murray, S.S. (2010). Brain-derived neurotrophic factor promotes central nervous system myelination via a direct effect upon oligodendrocytes. Neurosignals *18*, 186–202.

Xu, X., Stoyanova, E.I., Lemiesz, A.E., Xing, J., Mash, D.C., and Heintz, N. (2018). Species and cell-type properties of classically defined human and rodent neurons and glia. eLife *7*, e37551.

Zonouzi, M., Berger, D., Jokhi, V., Kedaigle, A., Lichtman, J., and Arlotta, P. (2019). Individual oligodendrocytes show bias for inhibitory axons in the neocortex. Cell Rep *27*, 2799–2808.e3.

STAR★METHODS

KEY RESOURCES TABLE

REAGENT or RESOURCE	SOURCE	IDENTIFIER
**Antibodies**		
Rabbit anti-OLIG2	Abcam	Cat#AB42453; RRID: AB_776905
mouse anti-NESTIN	Millipore	Cat#MAB5326; RRID: AB_2251134
m-Cy3 anti-GFAP	Sigma	Cat#C9205; RRID: AB_476889
mouse anti-NogoA	R&D systems	Cat#MAB3098; RRID: AB_10997139
goat anti-ChAT	Sigma	Cat#AB144P; RRID: AB_2079751
mouse anti-ISLET1/2	DSHB	Cat#39.4D5; RRID: AB_2314683
mouse anti-NEUN	Millipore	Cat#CLONE A60 MAB377; RRID: AB_2298772
mouse anti-MAP2	Sigma	Cat#M9942; RRID: AB_477256
rabbit anti-PV	SWant	Cat#PV27; RRID: AB_2631173
rabbit anti-SOX10	Abcam	Cat#AB155279; RRID: AB_2650603
rabbit anti-PDGFRα	CellSignaling	Cat#5241S; RRID: AB_10692773
rat anti-MBP	Abcam	Cat#AB7349; RRID: AB_305869
mouse anti-CNP	Atlas	Cat#AMAB91072; RRID: AB_2665789
chicken anti-NF-H	Biolegend	Cat#822601; RRID: AB_2564859
rabbit anti-CASPR	Abcam	Cat#AB34151; RRID: AB_869934
rabbit anti-CLAUDIN-11	Thermo Fisher Scientific	Cat#36-4500; RRID: AB_2533259
mouse anti-ANKYRIN G	UC Davis/NIH NeuroMab	Cat#clone N106/36; RRID: AB_2877524
chicken anti-Neurofascin	Thermo Fisher Scientific	Cat#PA5-47468; RRID: AB_2609964
**Chemicals, peptides, and recombinant proteins**		
Essential 8™ Medium	Thermo Fisher Scientific	Cat#A1517001
Matrigel Basement Membrane matrix	Scientific Laboratory Supplies	Cat#354230
Dispase	Thermo Fisher Scientific	Cat#17105041
Collagenase	Thermo Fisher Scientific	Cat#17104019
Iscove's Modified Dulbecco's Medium	Thermo Fisher Scientific	Cat#21980032
F-12 Nutrient Mix	Thermo Fisher Scientific	Cat#31765-027
SB- 431542	R&D Systems	Cat#1614/1
LDN-193189	Merck	Cat#SML0559
BSA	Europa Bioproducts	Cat#EQBAC62
Chemically Defined Lipid Concentrate	Thermo Fisher Scientific	Cat#11905031
Monothioglycerol	Sigma	Cat#M6145
Insulin	Sigma	Cat#11376497001
Transferrin	Sigma	Cat#10652202001
N-acetyl cysteine	Sigma	Cat#A8199
Antibiotic-Antimycotic	Thermo Fisher Scientific	Cat#15240062
Retinoic acid	Sigma	Cat#R2625
FGF-2	PeproTech	Cat#450-33
Purmorphamine	Sigma	Cat#540220
Advanced DMEM/F12	Thermo Fisher Scientific	Cat#12634028
N2 Supplement	Thermo Fisher Scientific	Cat#17502048
B27 Supplement	Thermo Fisher Scientific	Cat#17504044
GlutaMAX™ Supplement	Thermo Fisher Scientific	Cat#35050038
PDGF-AA	Peprotech	Cat#AF-100-13A
Smoothened agonist	Sigma	Cat#566660

(*Continued on next page*)

REAGENT or RESOURCE	SOURCE	IDENTIFIER
Continued		
IGF-1	PeproTech	Cat#AF-100-11
T3	Sigma	Cat#T6397
Heparin	Sigma	Cat#H3149
ITS	Thermo Fisher Scientific	Cat#51500-056
BDNF	R&D Systems	Cat#248-BD
Blebbistatin	Sigma	Cat#203390
Tetanus toxin	Sigma	Cat#T3194
Laminin	Sigma	Cat#L2020
Fibronectin	Sigma	Cat#F2006
Poly-L-ornithine	Sigma	Cat#P3655
Paraformaldehyde	Agar Scientific	Cat#AGR1026
Gluteraldehyde	Agar Scientific	Cat#R1010
Normal goat serum	Vector Laboratories	Cat#S1000
OCT-compound	Biosystems	Cat#14020108926
Toluidine blue	Wako Pure Chemical Industries	Cat#C.I.52040
Critical commercial assays		
RNeasy Mini Kit	Qiagen	Cat#74106
DyNAmo ColorFlash SYBR™ DyNAmo ColorFlash SYBR Green qPCR Kit	Thermo Fisher Scientific	Cat#10442308
Worthington Papain Dissociation System	Worthington-biochemical	Cat#LK003150
Epoxy resin Kit	Agar Scientific	Cat#Araldite CY212
Experimental models: cell lines		
CS02iCTR-n1	Cedars Sinai	N/A
CS25iCTR-18n2	Cedars Sinai	N/A
SHEF-4 (AXORe004-A)	UK Stem Cell Bank	N/A
CS00iNK-n1 and -n2	Chandran lab	N/A
Oligonucleotides		
Primers for Nfasc155 mutation site F: CACCCTTTTGTCCTGAGCCTT	This paper	N/A
Primers for Nfasc155 mutation site R: ATAGCCCGCATTTGCTACCC	This paper	N/A
qPCR primer for 18s F: GTAACCCGTTGAACCCCATT	This paper	N/A
qPCR primer for 18s R: CCATCCAATCGGTAGTAGCG	This paper	N/A
qPCR primer for LHX2 F: ATGCTGTTCCACAGTCTGTCG	This paper	N/A
qPCR primer for LHX2 R: GCATGGTCGTCTCGGTGTC	This paper	N/A
qPCR primer for OTX2 F: AGAGGACGACGTTCACTCG	This paper	N/A
qPCR primer for OTX2 R: TCGGGCAAGTTGATTTTCAGT	This paper	N/A
qPCR primer for HOXB4 F: GTGAGCACGGTAAACCCCAAT	This paper	N/A
qPCR primer for HOXB4 R: CGAGCGGATCTTGGTGTTG	This paper	N/A
qPCR primer for HOXB5 F: CGGGTCAGGTAGCGATTG	This paper	N/A
qPCR primer for HOXB5 R: AGCTTCACATCAGCCACGAT	This paper	N/A

(*Continued on next page*)

Continued

REAGENT or RESOURCE	SOURCE	IDENTIFIER
qPCR primer for HOXB8 F: CAGCTCTTTCCCTGGATGC	This paper	N/A
qPCR primer for HOXB8 R: ATAGGGATTAAATAGGAACTCCTTCTC	This paper	N/A
Software and algorithms		
Molecular Devices MetaXpress® High-Content Image Acquisition and Analysis Software	Molecular Devices	https://moleculardevices.com/products/cellular-imaging-systems/acquisition-and-analysis-software/metaxpress
R	https://www.R-project.org/	N/A
Fiji	https://imagej.net/Fiji	N/A
Other		
Millicell Cell Culture Insert	Merck	Cat#PICM0RG50
Superfrost Plus slides	VWR	Cat#631-0108

RESOURCE AVAILABILITY

Lead contact

Further information and requests for resources and reagents should be directed to and will be fulfilled by the Lead Contact, Siddharthan Chandran (Siddharthan.Chandran@ed.ac.uk).

Materials availability

Unique material generated in this study is available from the Lead Contact with a completed Materials Transfer Agreement.

Data and code availability

Relevant data and code are available from the corresponding author (SC) upon request.

EXPERIMENTAL MODEL AND SUBJECT DETAILS

Culture of human iPSCs

The human pluripotent stem cell-lines used in this study were obtained with full Ethical/Institutional Review Board approval by the University of Edinburgh and validated using standard methods including chromosomal analysis, pluripotency and absence of plasmid integration. The iPSC lines CS02iCTR-NTn1 (hPSC1, male) and CS25iCTRL-18n2 (hPSC2, male) were obtained from Cedars-Sinai. For the Nfasc155$^{-/-}$ lines, fibroblasts were obtained by RS with ethical approval granted by the Institutional Review Board of Warsaw Medical University (Smigiel et al., 2018). The CS00iNK-n1 (Nfasc155$^{-/-}_{clone\ 1}$, female) and CS00iNK-n2 (Nfasc155$^{-/-}_{clone\ 2}$, female) iPSC lines were generated by Cedars-Sinai. The human embryonic stem cell-line SHEF4 (hPSC3, male) was obtained from the UK Stem Cell Bank. iPSCs were maintained on Matrigel (Scientific Laboratory Supplies)-coated 6-well plates in Essential 8 medium (Thermo Fisher Scientific) at 37 °C and 5% CO2. iPSC colonies were passaged by incubating in Dispase (0.5 mg/ml, Thermo Fisher Scientific)/Collagenase (1 mg/ml, Thermo Fisher Scientific) for 15-25 minutes at 37 °C, washed in DPBS and resuspended in Essential 8 medium before being redistributed in new 6-well plates. Cultures were regularly tested and maintained mycoplasma free. These cell-lines have not been authenticated.

METHOD DETAILS

Generation of iPSC myelinoids

iPSC myelinoids were generated by adapting a previously published protocol for the generation of iPSC-derived oligodendrocytes (Livesey et al. 2016). Briefly on day 0, iPSC colonies were lifted using Dispase (0.5 mg/ml)/Collagenase (1 mg/ml), washed in DPBS and transferred to 10 cm dishes to be neuralized as a suspension culture in phase I medium (0.5 x Iscove's modified Dulbecco's medium (Thermo Fisher Scientific), 0.5 x F12 (Thermo Fisher Scientific), 20 μM SB- 431542 (R&D Systems) and 0.1 μM LDN-193189 (Merck), 5 mg/ml BSA (Europa Bioproducts), 1 x chemically defined Lipid concentrate (Thermo Fisher Scientific), 4.32 μg/ml monothioglycerol (Sigma), 7 mg/ml insulin (Sigma), 15 mg/ml transferrin (Sigma), 1 x Antibiotic-Antimycotic (Thermo Fisher Scientific) and 1 mM N-acetyl cysteine (Sigma)). On day 7, 1 μM retinoic acid (RA, Sigma) was added to pattern neurospheres toward a caudal fate (phase II). On day 14, neurospheres were plated onto laminin-coated dishes to selectively enrich for neural precursor cells (NPCs) by assessing neural rosette formation. On day 17, adhered

NPCs were lifted, returned to 10 cm dishes, and further differentiated in suspension using phase III medium containing FGF-2 (10 ng/ml, PeproTech) and 1 μM purmorphamine (Sigma) in N2/B27 base medium (Advanced DMEM/F12 (Thermo Fisher Scientific), 1 x N2 (Thermo Fisher Scientific), 1 x B27 (Thermo Fisher Scientific), 1 x Glutamax (Thermo Fisher Scientific), 1 x Antibiotic-Antimycotic (Thermo Fisher Scientific)) to expand and ventralize neural progenitor cells. After 7 days, neurospheres were transferred to phase III medium lacking FGF-2 and maintained for a subsequent 2 weeks to induce neuronal and glial differentiation. Next, neurospheres were transferred to oligodendrocyte proliferation medium (N2/B27 base medium plus 10ng/ml FGF-2 (PeproTech), 20 ng/ml PDGF-AA (PeproTech), 1 μM purmorphamine (Sigma), 1 μM smoothened agonist (SAG, Sigma), 10 ng/ml IGF-1 (PeproTech), 60 ng/ml T3 (Sigma), 1 x ITS (Thermo Fisher Scientific), 5μg/ml heparin (Sigma)). After 2-4 weeks, myelin induction (MI) was initiated by transferring individual spheroids (700-1500 μm diameter) onto PTFE-coated Millicell Cell Culture Inserts (Merck) in 6 well plates using a P200 pipette and maintained in myelination medium thereafter (N2/B27 base medium, 5 μg/ml heparin, 1 x ITS, 10 ng/ml IGF-1, 60 ng/ml T3) at 7.5 % CO_2, replacing the medium every 2-3 days. To modulate myelin sheath number per cell, blebbistatin (10 μM, Sigma) or TeNT (3 nM, Sigma) were added to myelination medium at MI-0 and maintained throughout the remainder of the culture period. To modulate global myelination, BDNF (10 ng/ml, R&D Systems) was added to the medium between MI-0 and MI-12 and the same TeNT-treated spheres were used as for single cell analysis. Four, eight or twelve weeks after being placed on cell culture inserts, myelinoids were fixed by immersing inserts in 4 % paraformaldehyde (PFA, Agar Scientific) for 2 hours at room temperature (RT) before being washed in PBS (2 x 30 minutes, RT) and kept at 4 °C before further processing.

Dissociated myelinoid cultures

MI-0 myelinoids were dissociated using the Worthington Papain Dissociation System (LK003150) as described previously (Magnani et al., 2019; Vasistha et al., 2019). Cells were plated at 20-30,000 cells per 0.3cm on coverslips coated with 1/100 diluted Matrigel (BD Biosciences), 20 μg/ml Fibronectin (Sigma), and 10 μg/ml Laminin (Sigma) and cultured with the same myelination medium as above. TeNT (3nM) was supplemented in the media from day 1 onwards. Cells were fixed at day 14 by immersing in 4 % PFA for 20 minutes at RT, before being washed in PBS and stained for MBP and DAPI.

Immunostaining of whole-mounted myelinoids

Cell counts, manual tracing of myelin sheaths per cell and automated measurements of myelin volume were performed on whole-mounted myelinoids. For immunostaining, membranes containing myelinoids were cut out and transferred to a 24-well-plate containing PBS. Cells were permeabilized in 0.25 % triton-X-100 in PBS for 40 minutes and blocked in blocking solution (10 % normal goat serum (Vector Laboratories) + 0.25 % triton-X-100) for 2 hours at RT. CNP immunostaining required an additional step of antigen retrieval by incubating myelinoids in citrate buffer (pH 6) at 95 °C for 20 minutes followed by a further hour in blocking solution. Primary antibodies were diluted in blocking solution and incubated overnight at 4 °C. We used rabbit anti-OLIG2 (Abcam, 1:200), mouse anti-NESTIN (Merck, 1:300), Cy3 conjugated anti-GFAP (Sigma, 1:500), mouse anti-NogoA (R&D systems, 1:100), goat anti-ChAT (Millipore, 1:250), mouse anti-Isl1/2 (DSHB, 1:100), mouse anti-NeuN (Millipore, 1:500), mouse anti-MAP2 (Sigma, 1:200), rabbit anti-PV (SWANT, 1:500), rabbit anti-SOX10 (Abcam, 1:500), rabbit anti-PDGFRα (Cell Signalling, 1:200), rat anti-MBP (Abcam, 1:100), mouse anti-CNP (Atlas, 1:2000), chicken anti-NF-H (Biolegend, 1:10,000), rabbit anti-CASPR (Abcam, 1:1000), rabbit anti-CLAUDIN-11 (Thermo Fisher, 1:75), mouse anti-ANKYRIN G (UC Davis/NIH NeuroMab, 1:100), chicken anti-Neurofascin (Thermo Fisher, 1:100). The next day, myelinoids were washed (3 x 20 minutes) in PBS + 0.1 % Tween-20 (PBS-T). Secondary antibodies were diluted in blocking solution and incubated for 2 hours at RT. We used Alexa Fluor goat anti-mouse IgG1, goat anti-mouse IgG2b, goat anti-rabbit IgG, goat anti-chicken IgY, donkey anti-goat IgG, donkey anti-rat and donkey anti-rabbit (all from Abcam, all 1:1000). Myelinoids were incubated with DAPI (Sigma, 1:50,000) for 10 minutes, washed in PBS and mounted onto microscope slides (Thermo Scientific) with FluorSave (Calbiochem) and No. 1.5 coverslips (Thermo Scientific).

Cryopreservation and immuno-staining

To assess myelin distribution, ensheathment of different neuronal subtypes and PNJ and nodal assembly, membranes containing myelinoids were cut out and incubated in 30 % sucrose in PBS at 4 °C for at least 24 hours. Next, myelinoids were transferred into moulds containing a 1:1 solution of 30 % sucrose and OCT-compound (Biosystems) and cryopreserved by placing moulds in a bath of isopentane over dry ice. 10 μm cryosections were cut using a Leica cryostat, mounted onto superfrost-plus glass slides (VWR International) and stored at -20 °C. Immuno-staining was performed by bringing slides to RT and washing in PBS (3 x 10 min). Sections were permeabilised in 0.25 % triton-X-100 for 15 minutes before being blocked in blocking solution for 2 hours. Primary antibodies (see above) were diluted in blocking solution and incubated in a humid chamber overnight at RT. Sections were washed in PBS-T (3 x 10 min) and incubated with secondary antibodies diluted in blocking solution for 2 hours at RT. Sections were stained with DAPI for 5 minutes, washed in PBS and mounted with FluorSave.

Image acquisition

A Zeiss 710 confocal was used for the following: for cell counts, two z-stacks per myelinoid (whole-mounted) were acquired with a 40X (1.3 NA) objective; for manual tracings of individual oligodendrocytes, a 40X objective was used to create tiled z-stacks (typically 3x4 tiles, 20 μm z-depth with 0.25 μm z-steps), and a minimum of 5 cells per myelinoid were measured.

CellPress
OPEN ACCESS

To acquire PNJs, a 63X oil objective (1.4 NA) was used. To acquire whole-mounted myelinoids for automated analysis of myelin volume, an ImageXpress micro confocal was used. Low magnification (4X) scans of each microscope slide were taken, and acquisition areas were drawn around each myelinoid. 12 μm z-stacks (1 μm z-step) beginning at the superficial edge of each acquisition area were acquired using a 20X plan Apo objective, in 42 μm pin hole confocal mode, and with 10% overlap for stitching. A custom macro was written in Fiji/ImageJ to stitch images together using the Grid/Collection stitching plugin (Preibisch et al., 2009).

Transmission electron microscopy

To demonstrate myelin compaction, myelinoids were fixed in phosphate buffer (PB) containing 4 % PFA and 2 % glutaraldehyde (Agar Scientific) for 2 hours, post-fixed in 0.1 % glutaraldehyde for 24 hours at 4 °C and washed in 0.1 M PB (2 x 15 min). Samples were transferred to 1 % OsO_4 in 0.1 M PB for 45 min at RT, washed in 0.1 M PB and then dehydrated in increasing concentrations of EtOH for 15 minutes each (50 %, 70 %, 90 %, 100 % x 3). The Epoxy Resin (Araldite) kit (Agar Scientific) was used for embedding samples. Briefly, samples were incubated in a 1:1 mix of Araldite-DDSA:acetone for 1 hour then transferred to Araldite-DDSA for overnight incubation before being cured in Araldite embedding mix at 60 °C for 48 hours. For toluidine blue staining, 1 μm semithin sections were cut, placed on Polysine slides (VWR) and flattened by rotating the slide on a heat plate. The slide was then flooded with toluidine blue (Wako, 5% in a Borax solution) and heated again until vapours evolved. The slides were washed in water, mounted with FluorSave and imaged on a Zeiss Observer microscope. For Transmission electron microscopy, ultrathin sections, 60 nm thick, were cut, stained in Uranyl Acetate and Lead Citrate then viewed using a JEOL JEM-1400 Plus TEM. Images were captured on a GATAN OneView camera.

PCR amplification of Nfasc155

To confirm the presence of the homozygous *NFASC* mutation in patient-derived iPSCs, primers flanking the rs755160624 mutation site were generated (F: 5' CACCCTTTTGTCCTGAGCCTT 3'; R: 5' ATAGCCCGCATTTGCTACCC 3') and PCR products from Nfasc155$^{-/-}$ and Ctrl iPSCs were sent for Sanger Sequencing (SourceBioscience).

Quantitative RT-PCR

qRT-PCR was performed according to a previously described protocol (Bilican et al., 2014). Briefly, 250ng of total RNA collected from MI-0 myelinoids using an RNeasy Mini Kit (Qiagen) was used to synthesise cDNA using the DyNAmoTM cDNA synthesis kit (Thermo Fisher Scientific). Real-time quantitative PCR reactions were prepared using the DyNAmo ColorFlash SYBR Green qPCR kit (Thermo Fisher Scientific) and performed on a CFX96 system (BioRad). The primers used for Figure 1D are as follows:

18s F: GTAACCCGTTGAACCCCATT, 18s R CCATCCAATCGGTAGTAGCG: LHX2 F: ATGCTGTTCCACAGTCTGTCG, LHX2 R: GCATGGTCGTCTCGGTGTC, OTX2 F: AGAGGACGACGTTCACTCG, OTX2 R: TCGGGCAAGTTGATTTTCAGT, HOXB4 F: GTGAGCA CGGTAAACCCCAAT, HOXB4 R: CGAGCGGATCTTGGTGTTG, HOXB5 F: CGGGTCAGGTAGCGATTG, HOXB5 R: AGCTTCACATCAG CCACGAT, HOXB8 F: CAGCTCTTTCCCTGGATGC, HOXB8 R: ATAGGGATTAAATAGGAACTCCTTCTC. Data was obtained by pooling 5 myelinoids per batch-conversions (n=3 batch-conversions per cell-line). 1/ΔCt values were obtained by normalizing to 18S rRNA expression levels.

QUANTIFICATION AND STATISTICAL ANALYSIS

Image analysis

Cell counts were performed using the built-in FIJI plugin, Cell Counter. Manual tracing of myelin sheaths was performed using the Simple Neurite Tracer plugin (Longair et al., 2011). Overlaying manual tracings of myelin sheaths with CASPR immuno-staining from the same region of interest confirmed we were accurately measuring sheath length (Figure 5B). Nearest neighbor analysis was performed to determine oligodendrocyte density by plotting a 100 μm radius circle around individual cells and counting the number of myelinating oligodendrocytes within that area (Figure S5A). Sholl analysis on MBP$^+$ cells from dissociated myelinoids was performed by thresholding each cell in Fiji and using the Sholl analysis plugin (Ferreira et al., 2014). All counts and measurements were performed blinded.

Automated analysis of myelin volume

To measure global myelin volume in 3D, a custom module was designed using the MetaXpress software from Molecular Devices. First, an adaptive threshold was used to segment either MBP or CNP immuno-staining. The Filter Mask tool was then used to remove objects with a Fiber Breadth > 16 μm, a Fiber Length < 10 μm and an Area < 45 μm^2. Objects were then shrunk by 4 pixels and a subsequent filter step removed objects that had an Elliptical Form Factor < 3.7, Fiber Breadth of > 8 μm and an Intensity Standard Deviation < 1000. Keep Marked Objects was then used to retain objects from the first Filter Mask that overlaid with the second Filter Mask of shrunk objects. This step specifically helped remove non-myelin objects including cell bodies and debris. The Find Blobs tool was used to further identify non-myelin objects with an approximate width between 8-100 μm, which were then enlarged by 5 pixels and subtracted from the Filter Mask. This step was repeated with blobs approximately 4-6 μm in width. Next, a 3D filter mask was obtained by connecting neighbouring z-steps using the Connect Touching Objects tool. A final filter step removed 3D objects with a volume ≤ 450 μm^3 and a diameter ≤ 45 μm. In order to measure NF-H intensity, a simple threshold was performed to segment the total image area, and Connect by Touching was used to segment the total image volume. The volume of segmented myelin (μm^3) and

the integrated intensity of NF-H$^+$ immuno-staining were measured within the total image volume, summated for each acquisition site. Downstream analysis was performed using R-studio to process exported data tables, aggregate measurements for each myelinoid and normalise data to a batch control.

Statistical analysis

Unless otherwise indicated, data are presented as boxplots showing the medians, interquartile ranges and Tukey-style whiskers that extend to 1.5 times the interquartile range. Statistical analysis was performed using R-studio and the generalised mixed models plugin glmmTMB (Brooks et al., 2017). For analysis of myelin sheath number and length per cell, nearest neighbour and Sholl analysis, n = individual cells. For cell counts and global analysis of myelin volume, n = individual myelinoids. Analysis of the proportion of mature and myelinating oligodendrocytes over time, myelin sheath length and myelin volume over time was performed with time-point as the fixed effect. Where n = individual cells, individual myelinoids were included as a random effect (with random intercepts) as 'unique myelinoid ID' to accommodate for clustering of data (Aarts et al., 2014). Analysis of sheath number per cell, mean sheath length per cell, nearest neighbour (oligodendrocyte density) and Sholl analysis were performed with treatment conditions as fixed effects and unique myelinoid ID, batch-conversion and cell-line included as random effects. Automated analysis of myelin volume was performed with treatment as a fixed effect and each batch-conversion of iPSCs included as a random effect. For each experiment, appropriate statistical analyses were made based on the distribution, normality and type of data. Further details and n numbers for each experiment can be found in the corresponding figure legend. Statistical reporting includes the mean difference between groups or time-points, 95% confidence interval (95% CI) and p value *$p < 0.05$, **$p < 0.01$, ***$p < 0.001$.

Developmental Cell, Volume *56*

Supplemental information

iPSC-derived myelinoids to study myelin biology of humans

Owen G. James, Bhuvaneish T. Selvaraj, Dario Magnani, Karen Burr, Peter Connick, Samantha K. Barton, Navneet A. Vasistha, David W. Hampton, David Story, Robert Smigiel, Rafal Ploski, Peter J. Brophy, Charles ffrench-Constant, David A. Lyons, and Siddharthan Chandran

Figure S1: Characterisation and reproducibility of myelinoids, related to Figure 1

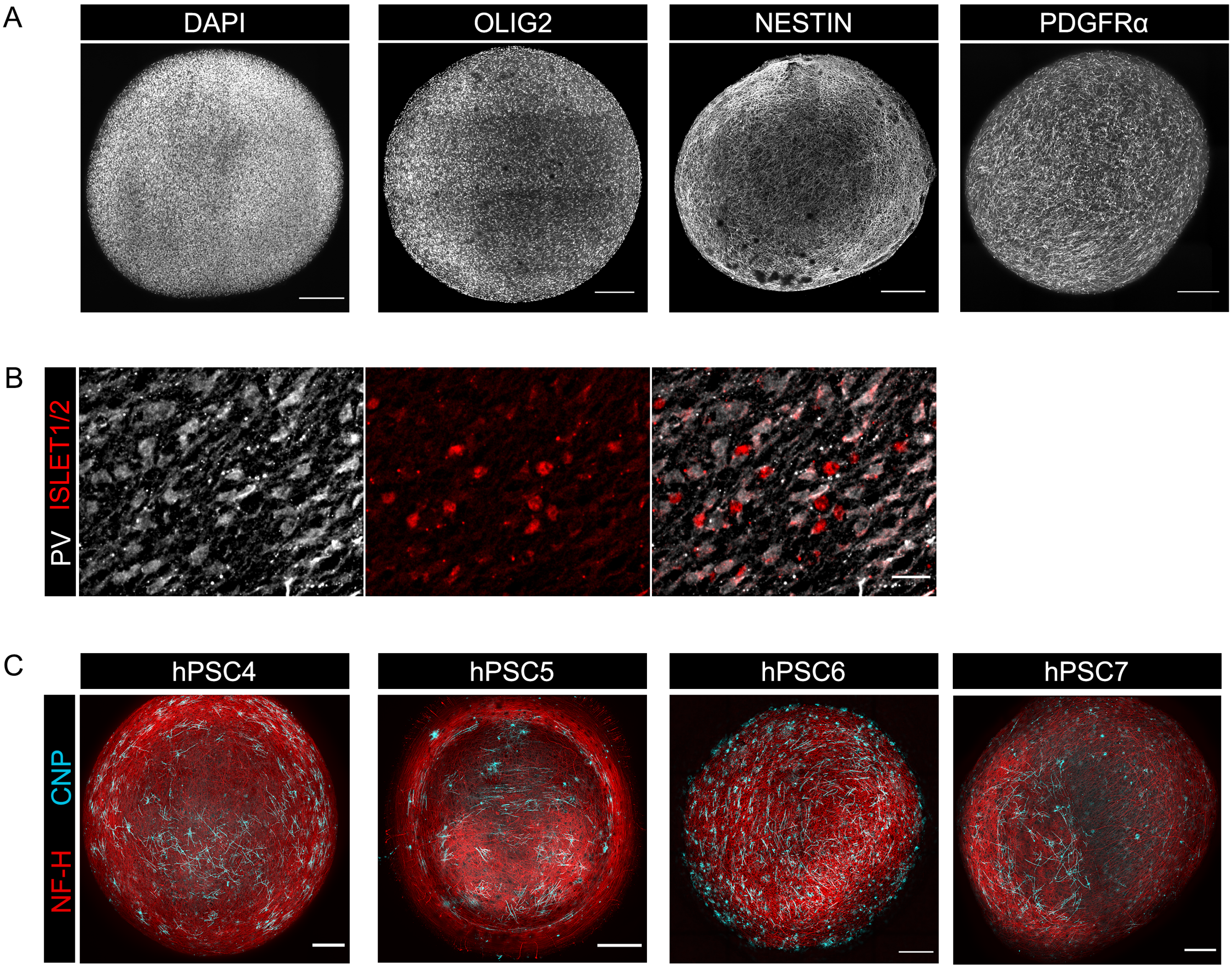

Figure S1: Characterisation and reproducibility of myelinoids, related to Figure 1.

A) Representative images of DAPI, OLIG2, NESTIN and PDGFRα at MI-0 in whole-mounted myelinoids (scale bar: 250μm).
B) Immuno-staining of PV and ISLET1/2 at MI-12 shows no colocalisation between these markers (scale: 25μm).
C) Representative images of CNP and NF-H-stained myelinoids at MI-12 from four additional cell-lines (scale bar: 250μm).

Figure S2: Distribution of myelin in iPSC myelinoids, related to Figure 2

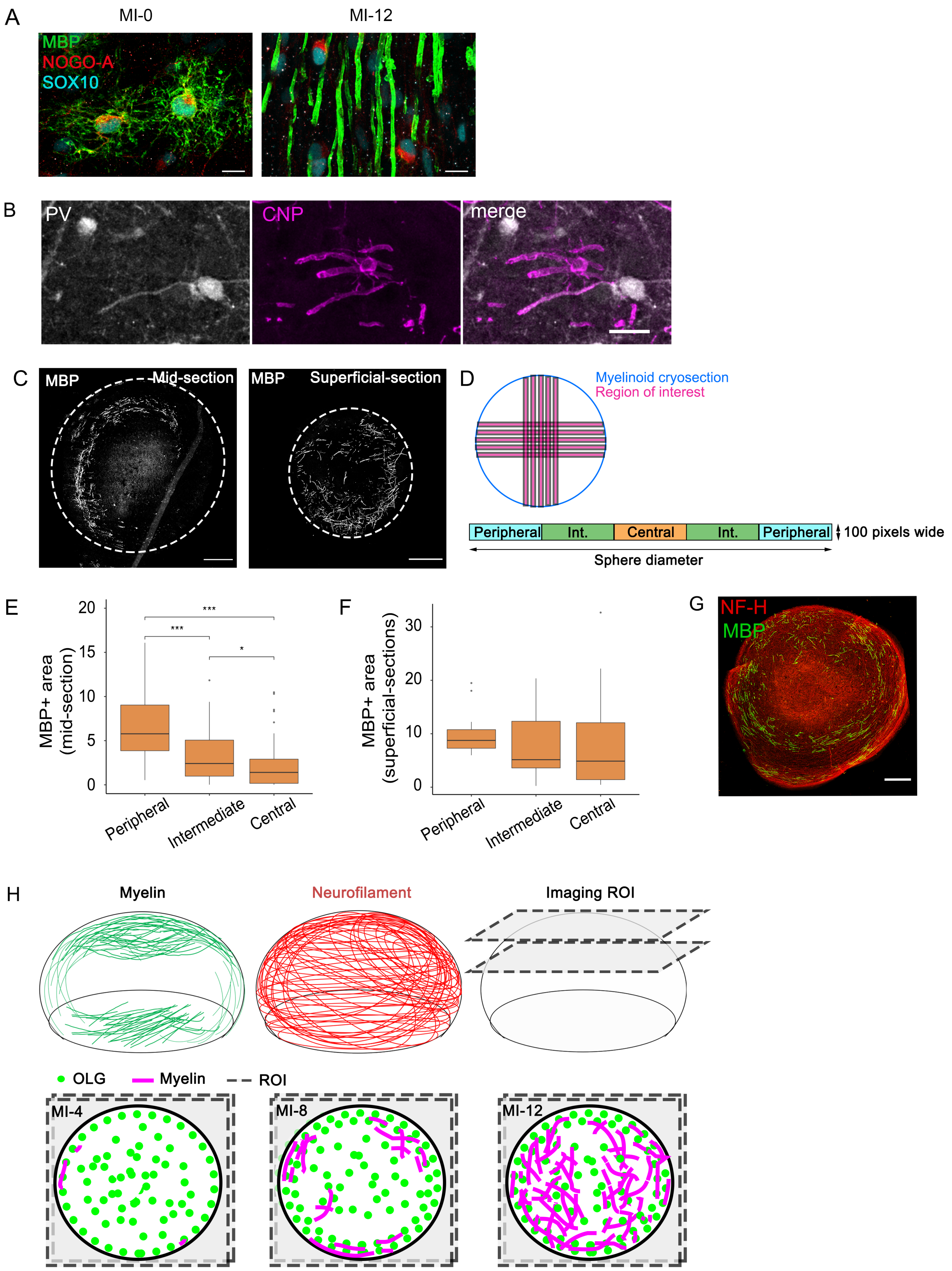

Figure S2: Distribution of myelin in iPSC myelinoids, related to Figure 2.

A) Mature oligodendrocytes stained for MBP, SOX10 and NOGO-A at MI-0 and MI-12 (scale bar: 10μm).
B) Immuno-staining of PV and CNP at MI-12 shows that individual oligodendrocytes can myelinate both PV+ and PV- axons simultaneously (scale bar: 25 μm).
C) Representative images of MBP+ myelin from a middle and superficial section of MI-12 myelinoids shows that myelin is predominantly found in the peripheral regions of mid-sectioned myelinoids and distributed across the entire area of the superficially-sectioned myelinoids (dashed line indicates section perimeter), scale bar: 250 μm.
D) Schematic overview for quantifying the distribution of MBP+ pixels across myelinoid cryosections. Multiple vertical and horizontal regions of interest were acquired across each section before images were thresholded and MBP+ pixels were measured as a function of the length of the ROI. Each ROI was then binned into peripheral, intermediate (Int.) and central regions for analysis (Figure S2E-F).
E-F) Quantification of MBP pixel area across middle and superficial myelinoid sections revealed 3.75-fold higher MBP+ pixel area in the periphery of sections compared to the central area (95% CI: 2.27- to 6.19-fold; $p < 0.001$); glmm with dummy variables for each region. n = 111 ROIs across 37 sections from 5 myelinoids from different batch-conversions.
G) Representative image of a mid-section myelinoid at MI-12 stained with MBP and NF-H shows myelination of axon tracts running around the periphery (scale bar: 250 μm).
H) Schematic depiction of the distribution of myelin and axons across iPSC myelinoids. The region of interest captured during whole-mounted imaging of myelinoids and schematic representation of myelin visualised within this ROI at MI-4, MI-8, and MI-12.

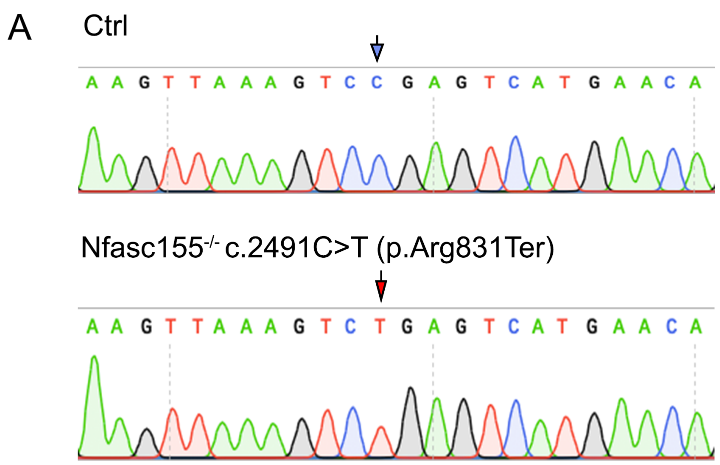

Figure S3: Sanger sequencing of NFASC gene, related to Figure 4.

A) Sanger sequencing of NFASC gene in control and Nfasc155-/- iPSCs, red arrow indicates nonsense mutation site.

Figure S4: Automated analysis of myelin distribution, related to Figure 5

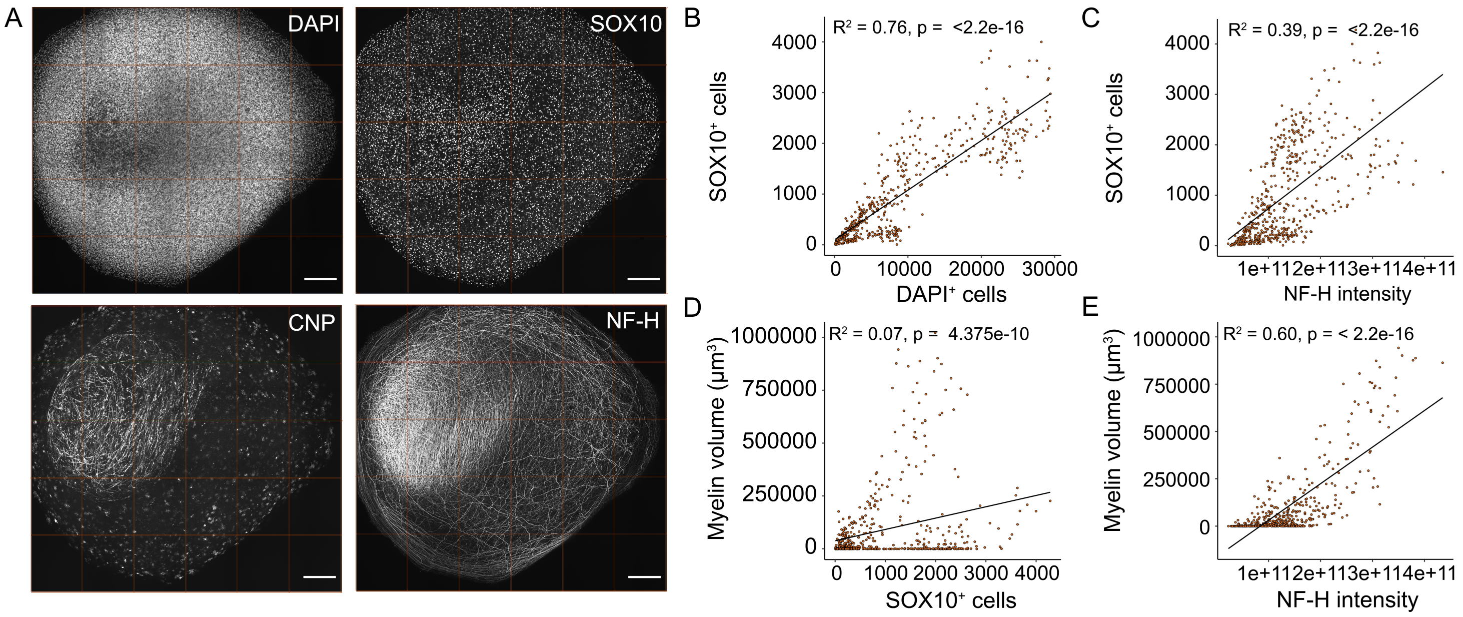

Figure S4: Automated analysis of myelin distribution, related to Figure 5.

A) Representative images of DAPI, SOX10, CNP and NFH staining from the same MI-12 myelinoid. Square grids indicate that each image is composed of 30 individual images tiled together. Correlation analysis performed on the small individual images was performed, scale bar: 250 µm.
B) Correlation of SOX10+ and DAPI+ cells, $R^2 = 0.76$, p = <2.2e-16.
C) Correlation of SOX10+ cells with NF-H intensity, $R^2 = 0.39$, p = <2.2e-16.
D) Correlation of myelin volume with SOX10+ cells, $R^2 = 0.07$, p = <4.38e-10.
E) Correlation of myelin volume with NF-H intensity, $R^2 = 0.6$, p = <2.2e-16.
Over 500 data points were analysed from 28 myelinoids at MI-12 across 6 conversions (Linear model with robust standard errors).

Figure S5: TeNT does not affect oligodendrocyte morphology cell-autonomously, related to Figure 6

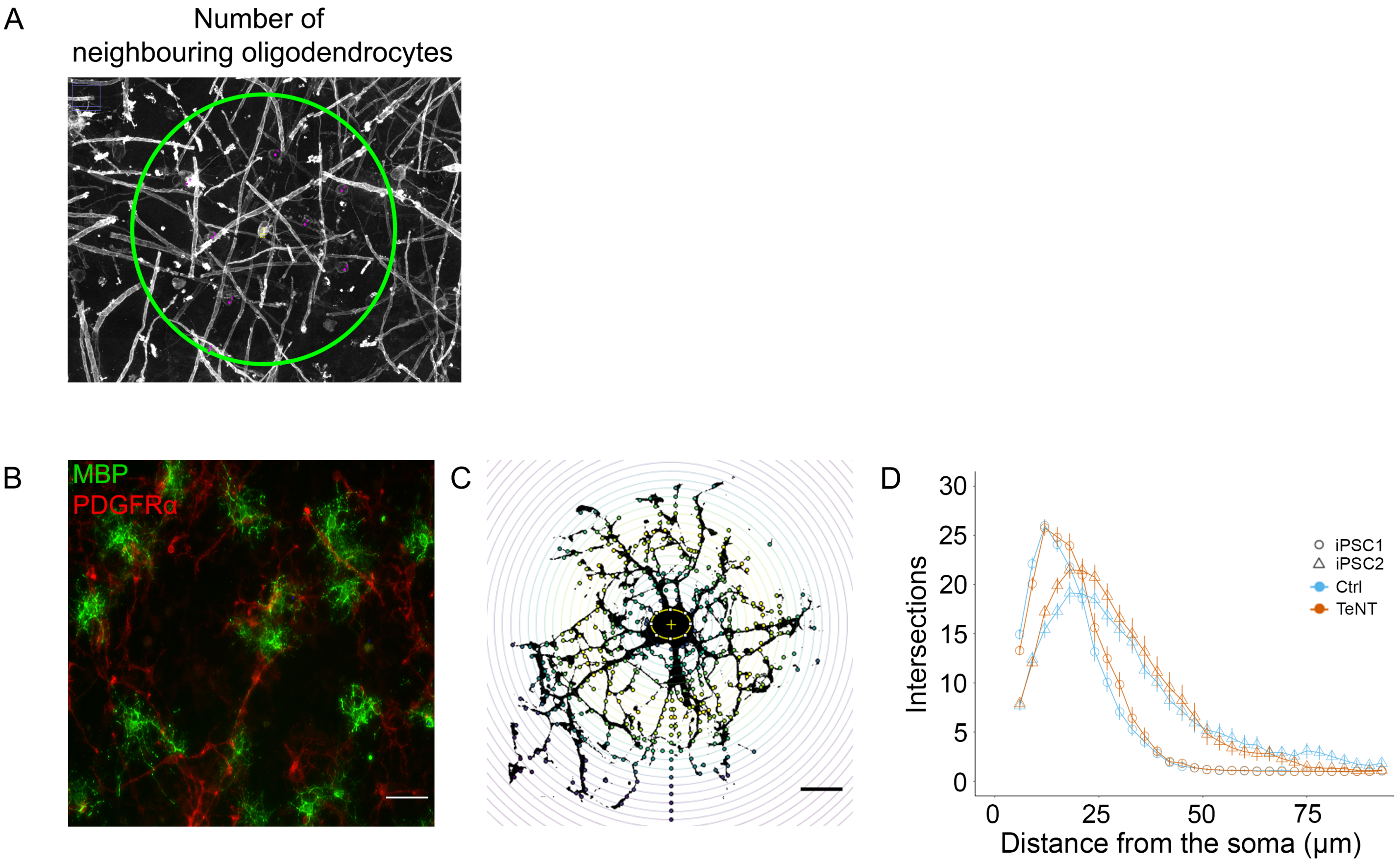

Figure S5: TeNT does not affect oligodendrocyte morphology cell-autonomously, related to Figure 6.

A) Nearest neighbour analysis was performed by drawing a 100 µm radius circle around individual cells and counting the number of myelinating oligodendrocytes within.
B) Representative images of MBP+ oligodendrocytes and PDGFRα+ OPCs from dissociated MI-0 myelinoids at day 14 (scale bar: 25µm).
C) Representative image of Sholl analysis on an MBP+ oligodendrocyte (scale bar: 10 µm; sholl radii emanating from the cell body (coloured) in 3 µm steps).
D) Sholl analysis of day 14 MBP+ oligodendrocytes from two different cell-lines treated with TeNT. Statistical modelling was performed as described by Wilson et al. (2018), J Neurosci Methods, glmm with radius and treatment as fixed effects and cell-line and batch-conversion as random effects. BH adjusted p values show no significant differences at any radii, n=84 cells (Ctrl) and 80 cells (TeNT) across two cell-lines and 3 batch-conversions each.
